# CrisprVi: a software for visualizing and analyzing CRISPR sequences of prokaryotes

**DOI:** 10.1186/s12859-022-04716-9

**Published:** 2022-05-11

**Authors:** Lei Sun, Jinbiao Wang, Fu Yan, Gongming Wang, Yun Li, Jinlin Huang

**Affiliations:** 1 School of Information Engineering, Yangzhou University, Yangzhou, People’s Republic of China; 2 School of Artificial Intelligence, Yangzhou University, Yangzhou, People’s Republic of China; 3 Jiangsu Key Laboratory of Zoonosis, Yangzhou, People’s Republic of China; 4 Beijing Institute of Genomics, Chinese Academy of Sciences, Beijing, People’s Republic of China; 5 Jiangsu Province Engineering Research Center of Knowledge Management and Intelligent Service, Yangzhou, People’s Republic of China; 6 Jiangsu Co-Innovation Center for Prevention and Control of Important Animal Infectious Diseases and Zoonosis, Yangzhou, People’s Republic of China

**Keywords:** CRISPR, Direct repeat, Spacer, Visualization, Statistics, Consensus sequence

## Abstract

**Background:**

Clustered regularly interspaced short palindromic repeats (CRISPR) and their spacers are important components of prokaryotic CRISPR-Cas systems. In order to analyze the CRISPR loci of multiple genomes more intuitively and comparatively, here we propose a visualization analysis tool named CrisprVi.

**Results:**

CrisprVi is a Python package consisting of a graphic user interface (GUI) for visualization, a module for commands parsing and data transmission, local SQLite and BLAST databases for data storage and a functions layer for data processing. CrisprVi can not only visually present information of CRISPR direct repeats (DRs) and spacers, such as their orders on the genome, IDs, start and end coordinates, but also provide interactive operation for users to display, label and align the CRISPR sequences, which help researchers investigate the locations, orders and components of the CRISPR sequences in a global view. In comparison to other CRISPR visualization tools such as CRISPRviz and CRISPRStudio, CrisprVi not only improves the interactivity and effects of the visualization, but also provides basic statistics of the CRISPR sequences, and the consensus sequences of DRs/spacers across the input strains can be inspected from a clustering heatmap based on the BLAST results of the CRISPR sequences hitting against the genomes.

**Conclusions:**

CrisprVi is a convenient tool for visualizing and analyzing the CRISPR sequences and it would be helpful for users to inspect novel CRISPR-Cas systems of prokaryotes.

**Supplementary Information:**

The online version contains supplementary material available at 10.1186/s12859-022-04716-9.

## Background

Prokaryotic (including half of bacterial and most archaeal) genomes contain CRISPR-Cas systems mainly composed of clustered regularly interspaced short palindromic repeats (CRISPR) array, the promoter for its transcription (the leader) and a set of CRISPR-associated (Cas) genes [1–3]. The CRISPR-Cas systems are formed by the fight against invasive genetic elements (e.g. phages and plasmids) during the evolution of prokaryotes, and they are sort of adaptive immunity that can block the infection process [4–6]. Due to the functional properties of the CRISPR-Cas systems, current research around them has expanded to a broad area, covering their origins and structures [7], prokaryotic population [8], CRISPR typing [9], gene editing [10, 11], etc.

As two key elements of the CRISPR-Cas system, direct repeats (DRs) of 24–50 bp and similar sized spacers between the DRs records what kind of RNA molecules can be derived from the array for activating the adaptive immunity [12]. Thus, it is important to investigate the loci and sequences of the DRs and spacers comprehensively. For a prokaryotic genome, it is straight forward to predict its CRISPR array/sequences using computational methods, such as CRT [13], PILER-CR [14], CRISPRFinder [15], CRISPRCasFinder [12], MinCED [16], CRISPRDetect [17], MetaCRAST [18], and CRISPRdisco [19]. And several CRISPR related databases, such as CRISPRdb [20], CRISPRI [21], CRISPRCasdb [22], also integrate programs for CRISPR identification, some of which are those mentioned above. However, there are few tools specifically designed to visualize, manipulate, and analyze the CRISPR arrays. In the past, Excel macro programs were commonly used to visualize the CRISPR sequences, but the programming is complicated and not interactive. In recent years, two computational tools, namely CRISPRviz [23] and CRISPRStudio [24], were developed for analyzing the CRISPR sequences interactively. For example, CRISPRviz can be used to predict, visualize and manipulate the CRISPR sequences by a web interface. Specifically, CRISPRviz can extract CRISPR DRs and spacers using MinCED [16], compare the sequence graphics visually and conduct alignment of spacer arrays. However, when the input includes a lot of strains with complex composition of CRISPR sequences, CRISPRviz will produce a variety of combinations of colors and symbols for the CRISPR sequences, which may be quite confusing. Unfortunately, CRISPRviz does not provide methods to help users analyze such complex scenario. Another problem of CRISPRviz is that it strongly depends on MinCED for CRISPR detection, which affects the accuracy of CRISPR visualization. Another tool, namely CRISPRStudio, can only present the spacers in a graphical way. Neither CRISPRviz nor CRISPRStudio provides comprehensive functions for the users to manipulate and analyze the DRs/spacers, such as changing colors of the visulization. For investigating and comparing the CRISPR loci of prokaryotic strains more intuitively and interactively, here we propose a novel visualization tool named CrisprVi.

## Implementation

### Framework of CrisprVi

**Fig. 1:**
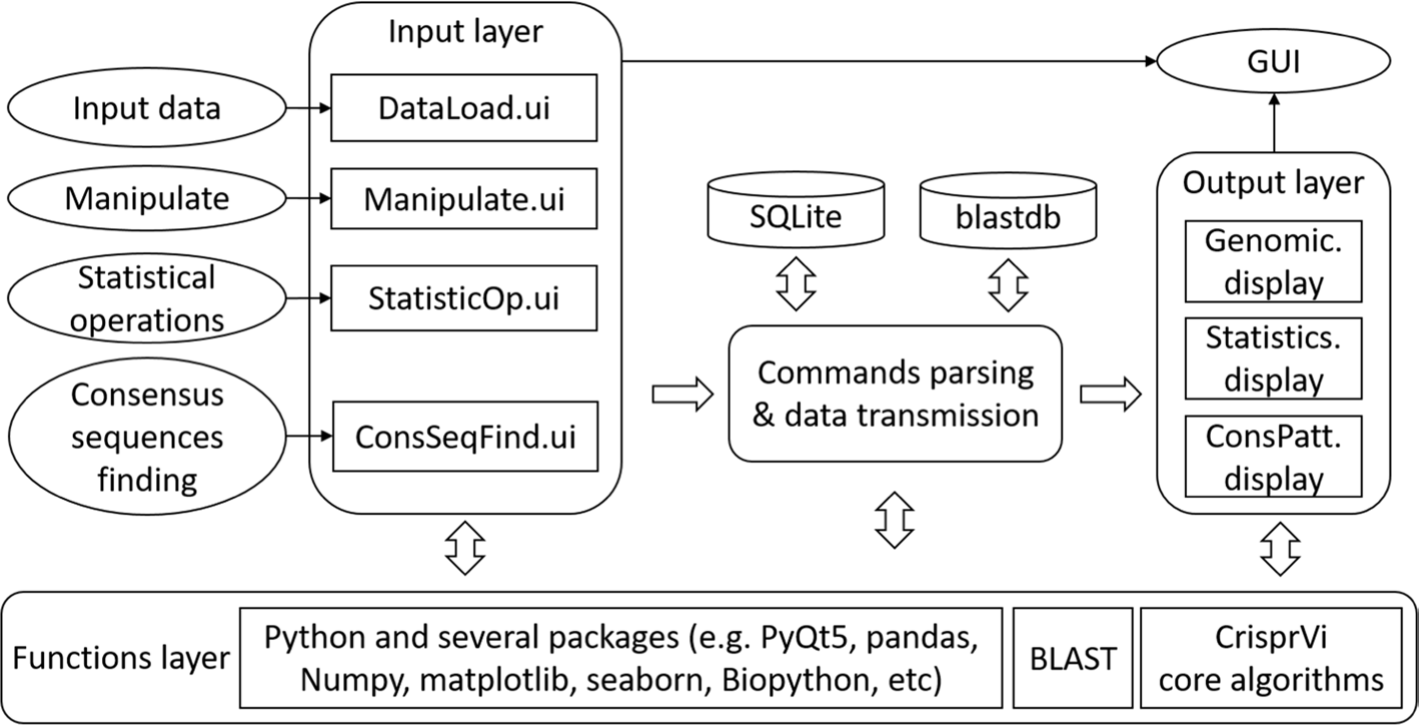
Software framework of CrisprVi. It mainly consists of a graphic user interface for visualization, a module for commands parsing and data transmission, local SQLite and BLAST databases for data storage and a functions layer supporting data processing

CrisprVi mainly consists of a graphic user interface (GUI) for visualization, a module for commands parsing and data transmission, local SQLite and BLAST [25, 26] databases for data storage and a functions layer supporting data processing (see Fig. 1). The GUI including input and output layers based on PyQt5 [27] is used to load, access and manipulate the CRISPR sequences interactively. The annotation data of CRISPR sequences in general feature format (GFF, see documentation of CrisprVi) [13] is extracted and stored in the SQLite database. And optionally raw genomes sequences can be converted to BLAST databases and be further used to find consensus sequences of DRs/spacers. The functions layer of CrisprVi is composed of CrisprVi core algorithms, BLAST and several Python packages, such as PyQt5, pandas [28], Numpy [29], matplotlib [30], seaborn [31], Biopython [32], etc. The pandas and Numpy packages are used for data processing and computation while the matplotlib and seborn packages are used to visualize results of the statistical analysis and consensus DR/spacer sequence finding.

### GUI

The GUI provides users with a convenient way to load the original data of CRISPR sequences, to present the data in various graphical ways, to manipulate the graphics, to conduct statistical analysis of the CRISPRs, and to find consensus DR/spacer sequences across strains.

The input layer consists of four modules, namely DataLoad.ui, Manipulate.ui, StatisticOp.ui and ConsSeqFind.ui, for the users to input data and operation commands through the graphic interface. To facilitate data loading, CrisprVi provides a wizard guiding the users to load GFF files and to set input/output directories step by step. First, the users need to load CRISPR annotation files in GFF, which contain all information of DRs and spacers of comparative strains, such as ID of the source genome, type (DR/spacer), coordinates on the genome, strand/direction, sequence and IDs. By default, the GFF file is the standard output by several CRISPR prediction programs, e.g. CRISPRCasFinder and CRISPRDetect. It is noting that the users should prepare the GFF files before starting CrisperVi. The CRISPR annotation information imported by the users will be stored in the SQLite database for subsequent query, delete, add and other operation.

After loading the GFF files of CRISPR annotations, the graphics of DRs and spacers will be shown in the main window by an output module Genomic.display, which includes three sub-modules, namely ‘DRs and Spacers’, ‘Spacers’ and ‘DRs’, for presenting CRISPR information in different ways. Then the users can directly manipulate the DRs and spacers by clicking on the graphics or specific buttons on the board provided by Manipulate.ui. The general manipulation includes showing CRISPR information (ID, source, start, end, strand, sequence, etc.), zooming in/out, deleting strains, sorting strains, aligning spacers, adding/deleting gaps.

Moreover, the users can conduct several statistical analysis on the CRISPR annotation data, such as counting DRs and spacers of strains, and calculating GC contents of DRs and spacers, etc., by commands provided by the module StatisticOp.ui. The results of the statistical analysis will be shown in figures by Statistics.display.

When the users are interested in the occurrences of consensus (identical or similar) sequences of DRs/spacers across strains, the module ConsSeqFind.ui can first find out the hits of DRs/spacers on the input genomes using BLAST, and then conduct clustering based on the BLAST results (see “Consensus sequences finding” section for details), which will generate a clustering heatmap through ConsPatt.display for the users to analyze the consensus patterns of CRISPR sequences.

### Commands parsing and data transmission

CrisprVi provides several functions to parse different operation commands sent from the users via GUI before triggering different functions, and the results will be returned and displayed by the output layer. Meanwhile, the data channels are used for data transmission between the caller and specific executive functions.

### Databases

The CRISPR information, such as *id*, *source*, *start*, *end*, *strand*, and *sequence*, in the input GFF files are extracted and stored in the SQLite database, where the users can add, delete, and search for the query sequences. Table 1 shows the format of the CRISPR database. On the other hand, the genome sequences of comparative strains will be converted to BLAST databases using commands provided by the BLAST package.

**Table 1:** Formats of properties of the CRISPR database

Field name	Datatype	Len	Not null?
id	varchar	150	No
source	varchar	150	No
type	varchar	20	No
start	int	50	No
end	int	50	No
sequence	varchar	150	No
strand	varchar	50	No

### CrisprVi core algorithms

To support several functions of interactive operation of the CRISPR sequences, we integrate several core algorithms in CrisprVi as follows.

#### Alignment of spacer arrays

It is a modified pairwise alignment method named SpacerAlign for comparing the similarity of spacer arrays of input strains. Specifically, it first constructs a diagonal score matrix by conducting pairwise alignment (the Needleman-Wunsch algorithm [33, 34]) on each pair of spacer arrays. Then the matrix is used to construct an UPGMA tree [35], which guides the alignment of multiple spacer arrays progressively.

#### Consensus sequences finding

It is an algorithm designed for detecting consensus sequences of DRs/spacers across input strains using local BLAST and clustering. In the module of consensus sequences finding, each DR/spacer of the input strains is searched for in the genome database using BLAST. Then a DR/spacer by genome matrix (DG or SG matrix) is constructed by a calculation method based on the BLAST results. Specifically, for each BLAST output file, the highest product of hit score and identity percentage (%) fills a cell of the DG/SG matrix corresponding to each pair of DR and genome. After that, the constructed DG/SG matrix is fed to the seaborn package for showing a clustering heatmap, from which the users can find some patterns of the CRISPR consensus sequences across strains.

#### Other algorithms

Several other algorithms for such as custom sequence extraction, SQLite data access and conversion and so on, are also included in the CrisprVi core algorithms.

### Test datasets

To evaluate CrisprVi and other CRISPR visualization tools, we first prepared dataset-I containing core genomes of 12 *Campylobacter coli* (*C*. *coli*) and 12 *Campylobacter jejuni* (*C*. *jejuni*) strains (see Additional file 1 for summary of dataset-I), which were downloaded from NCBI (https://www.ncbi.nlm.nih.gov/) [36] in March 2020. To evaluate the tools in visualizing large datasets, we prepared dataset-II containing 100 prokaryotic DNA sequences (see Additional file 2 for summary of dataset-II), which were downloaded from NCBI in Jun 2021. To conduct the comparison more fairly, we used two strategies as follows to obtain the CRISPR annotations. The strain counts of dataset-I and dataset-II are summarized in Table 2.

**Table 2:** Strain counts of dataset-I and dataset-II

Datasets	Dataset-I		Dataset-II
Species	*C. coli*	*C. jejuni*	Diverse
Original strain count	12	12	100
Strain count after annotation			
CRISPRCasFinder	12	12	N/A^1^
MinCED	9	8	80
MinCED to GFF	9	8	80

1. CRISPRCasFinder was not conducted on dataset-II

#### CRISPR annotation based on CRISPRCasFinder

The online tool CRISPRCasFinder [12] was used to generate GFF files containing CRISPR annotations for 12 *C. coli* and 12 *C. jejuni* of dataset-I respectively. Then the GFF files were input to CrisprVi for visualization and analysis. It should be noticed that the GFF files cannot be import to CRISPRviz, which only requires the FASTA files of genomes as input.

#### CRISPR annotation based on MinCED

On the other hand, we imported the genomes of dataset-I to CRISPRviz by running its *crisprviz.sh* script (with parameters -pxc), which calls MinCED [16] to annotate CRISPRs. It is noticed that MinCED just generated CRISPR annotation files for 9 *C. coli* and 8 *C. jejuni* strains on dataset-I, which were all converted to GFF files using the *minced2gff.py* script packaged in CrisprVi for further loading to CrisprVi (Table 2). On dataset-II, we used the same strategy based on MinCED to obtain 80 CRISPR annotation files, which were all converted to GFF files for inputting to CrisprVi.

## Results

### CrisprVi provides GUI for analyzing CRISPR sequences

**Fig. 2:**
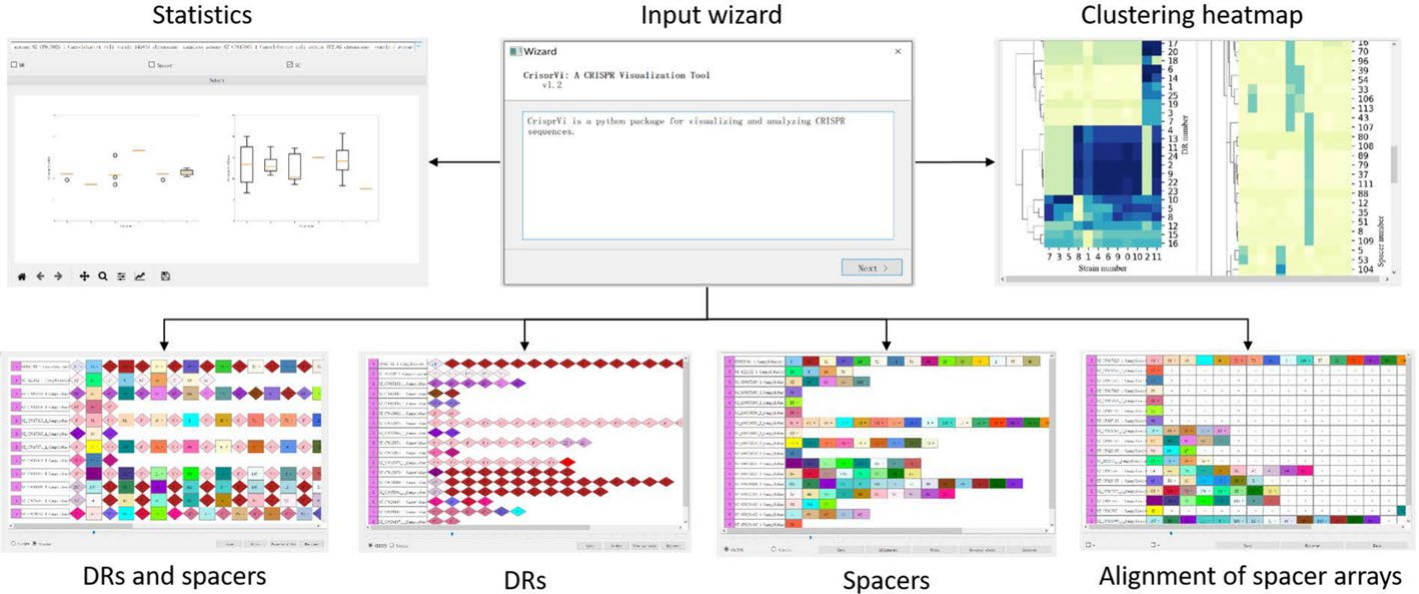
Modules of CrisprVi. CrisprVi contains several functional modules, such as data loading wizard (lower central), showing DRs and/or spacers (the three on top left), alignment of spacers (top right), statistics of DRs and/or spacers (lower left), and consensus patterns of DRs/spacers (lower right)

CrisprVi is a graphically interactive software including several function modules, such as showing DRs and/or spacers, alignment of spacers, statistics of DRs and/or spacers, and consensus patterns of DRs/spacers, as shown in Fig. 2. In comparison with other visualization tools such as CRISPRviz that can predict the CRISPR sequences using specific build-in tool, our CrisprVi just focuses on visualizing the CRISPR sequences of comparative strains extracted from the CRISPR annotations in GFF. Thus, CrisprVi can be more flexible as the users can choose any suitable tool to predict the CRISPR sequences before further visualization.

### Interactive operation of DRs and/or spacers

**Fig. 3:**
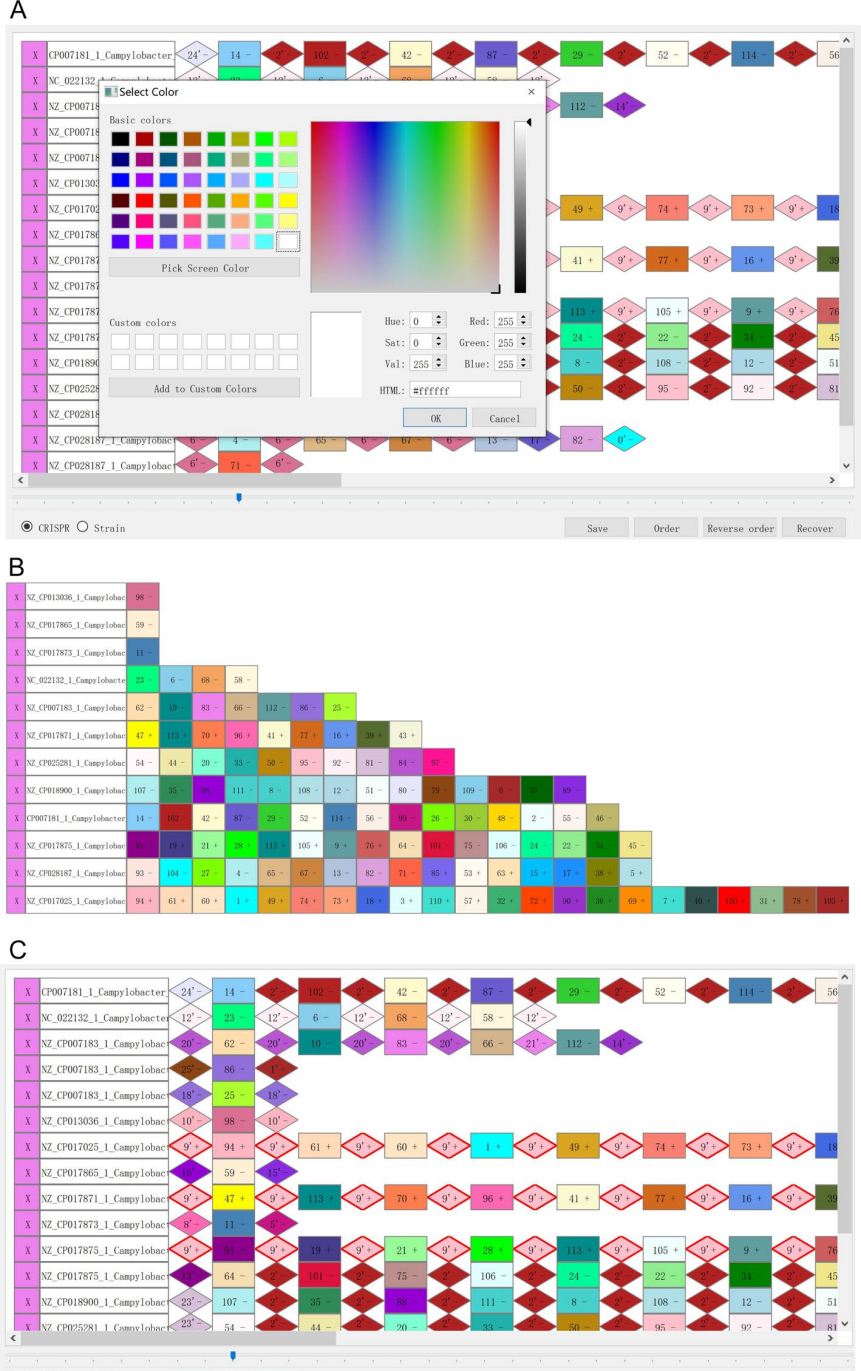
Illustration of interactive operation of CrisprVi. **A** Changing graphic color manually. **B** Sorting spacer arrays by length. **C** Highlighting identical CRISPRs by red boarders. In this figure, the visualized CRISPRs of 12 *C*. *coli* strains were predicted by CRISPRCasFinder

By clicking on different buttons/menus on the panel of CrisprVi, the users can change and manipulate the graphics denoting the DRs/spacers in several ways, such as showing DRs and spacers together or separately, and aligning spacer arrays (see Fig. 2). In CrisprVi, each spacer is denoted by a colored rectangular graphic with an inner number plus strand inside while the DR is represented by a colored diamond graphic with an inner number followed by an apostrophe plus strand inside. It is noting that the inner number is assigned to the DRs/spacers having the same sequence. To discriminate between different DRs/spacers visually, CrisprVi automatically assigns different combinations of colors and numbers for the DRs/spacers with different nucleotide composition. If the users do not like the color automatically assigned, they can right-click on the graphic and change the color by the color palette (see Fig. 3A). In the display area, the DRs and/or spacers of each strain or CRISPR is displayed in a track, which can be deleted selectively. And the users can sort the tracks according to their lengths (see Fig. 3B), namely the counts of DRs and/or spacers of the tracks. Once moving the mouse arrow onto specific DR/spacer, the information of the DR/spacer will be displayed over the graphic. Likewise, if the users click on the graphic, the information of the DR/spacer will be displayed on the panel under the scrollbar area, and all graphics having the same sequence as the selected one will be highlighted by red boarders (Fig. 3C), which can help the users view the global distribution of specific DR/spacer across strains more clearly.

### Statistical analysis of DRs and spacers

**Fig. 4:**
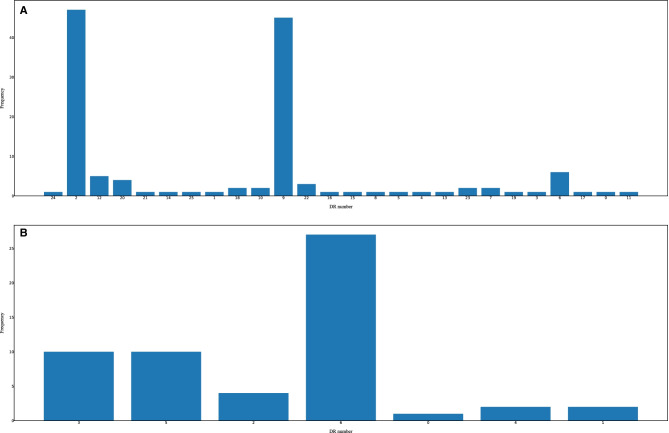
DR occurrences of the dataset-I strains. **A** DR occurences of 12 *C. coli* strains. **B** DR occurences of 12 *C. jejuni* strains. In this figure, the visualized statistics of DRs was predicted by CRISPRCasFinder

**Fig. 5:**
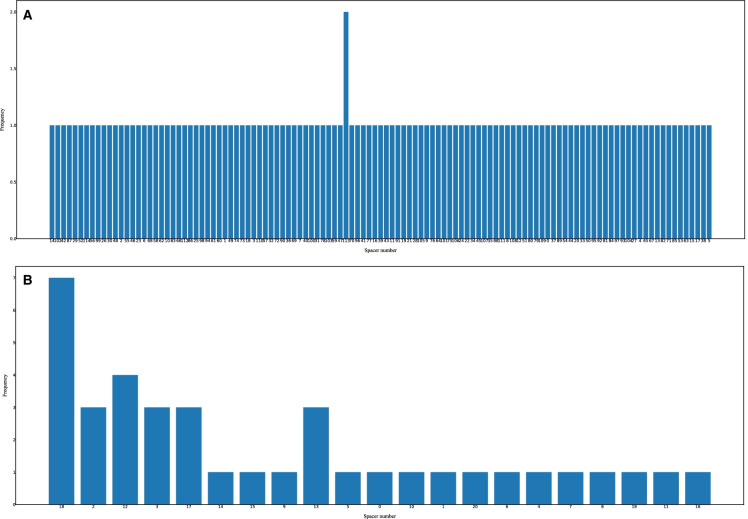
Spacer frequencies of the dataset-I strains. **A** Frequencies of *C. coli* spacers. **B** Frequencies of *C. jejuni* spacers. In this figure, the visualized statistics of spacers was predicted by CRISPRCasFinder

**Fig. 6:**
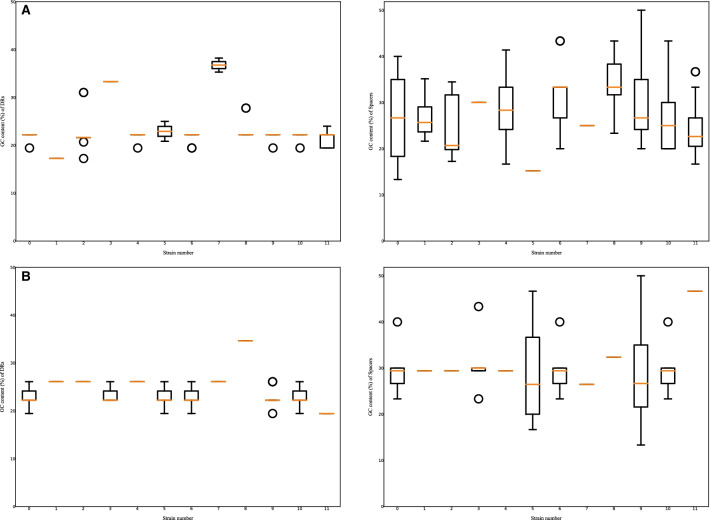
GC% distribution of CRISPRs of the dataset-I strains. **A** GC% distribution of CRISPRs of 12 *C. coli* strains. **B** GC% distribution of CRISPRs of 12 *C. jejuni* strains. In this figure, the visualized statistics of GC contents was predicted by CRISPRCasFinder

To obtain basic statistics of the DRs/spacers quickly, the users can perform statistical analysis of the DRs/spacers on the selected strains of interest (Figs. 4 and 5). First, the DRs occurred in the strains can be counted and displayed in histograms (see Fig. 4). As seen from the histogram in Fig. 4A, the repeat DR2 (type = ‘dr’, inner number = ‘2’) occurs the most frequently in all of the 12 *C*. *coli* strains while DR6 (type = ‘dr’, inner number = ‘6’) of *C*. *jejuni* is the most frequent repeat (Fig. 4B). The original source IDs of the DR with an inner number can be traced back by clicking on the graphics with the same inner number in other visualization modules mentioned previously. Second, the spacer counts across strains can be calculated and visualized in histograms, as shown in Fig. 5. Third, the users can compare the distribution of GC contents between DRs and spacers globally for the selected strains using boxplots (Fig. 6). In addition, the users can inspect details of the plots using commands in the menu under the plot. As seen from the statistical figures of the CRISPRs of comparative *C. coli* and *C. jejuni* strains from dataset-I, the *C. coli* strains represented more diverse properties than *C. jejuni* in terms of DRs/spacers categories and frequencies. Previous study also showed that the *C. coli* isolates presented more diverse allelic distribution than *C. jejuni* [37]. All these discoveries reveal the evolutionary differences between *C. coli* and *C. jejuni* strains.

### Analysis of consensus DR/spacer sequences

**Fig. 7:**
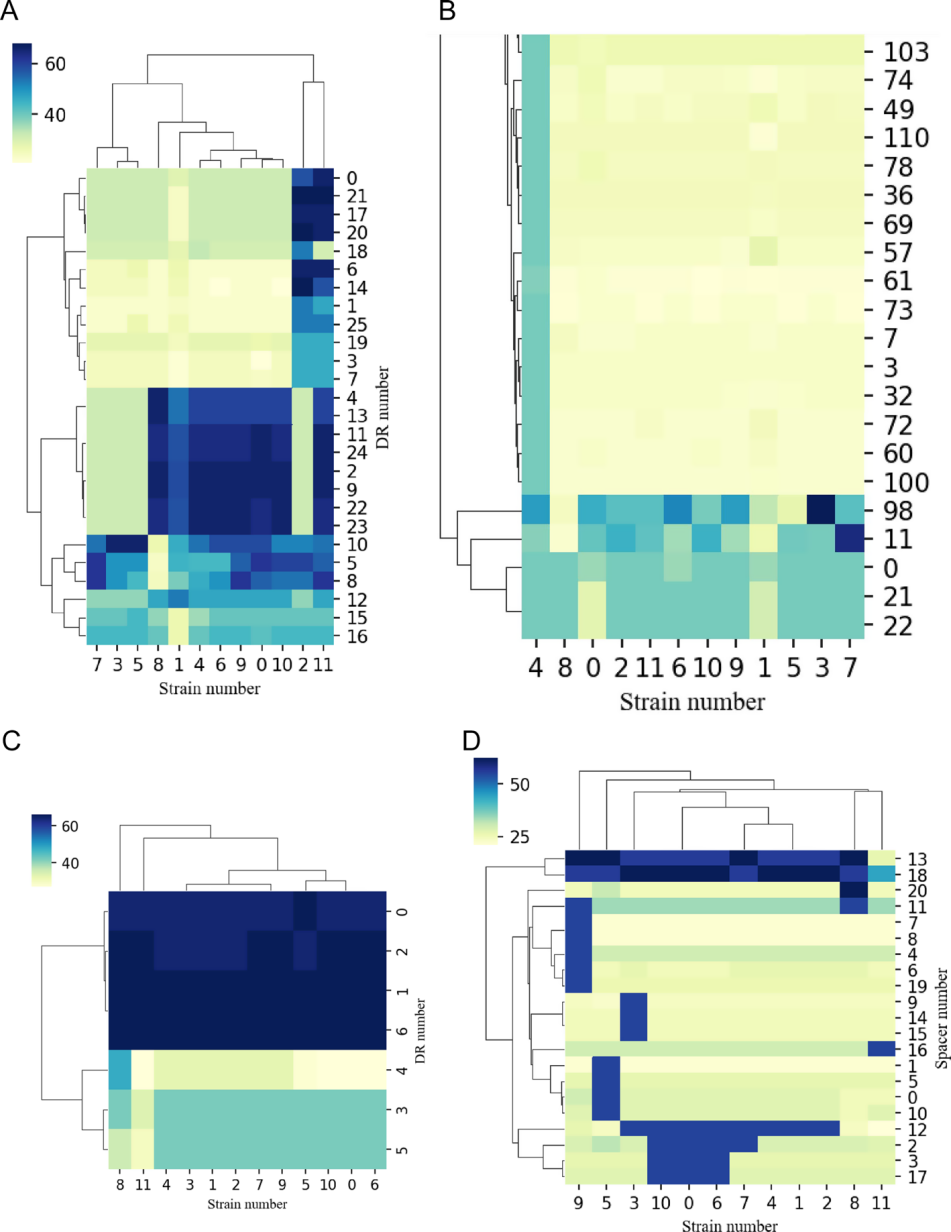
Pattern of consensus DR/spacer sequences of the dataset-I strains. **A** Pattern of consensus DR sequences of *C. coli*. **B** Pattern of consensus spacer sequences of *C. coli* (partial). **C** Pattern of consensus DR sequences of *C. jejuni*. **D** Pattern of consensus spacer sequences of *C. jejuni*. In this figure, the visualized pattern of consensus DR/spacer sequences was predicted by CRISPRCasFinder

CrisprVi provides a method for analyzing consensus sequences of DRs/spacers in all input genomes based on BLAST and clustering (see “Consensus sequences finding” section). Take dataset-I as an example, we first established local BLAST libraries for the genomes of *C. coli* and *C. jejuni* strains respectively, and then CrisprVi was used to align the DR/spacer sequences against the genomes in the libraries by calling the local BLAST. Based on the BLAST results, a DG/SG matrix was generated and visualized in a clustering heatmap, from which we can see how consensus each DR/spacer sequence occurs in the input strains, as shown in Fig. 7. First, the resulting heatmaps can be used to compare consensus sequence patterns across strains between DRs/spacers. For example, the DR sequences of the 12 *C*. *coli* strains represent a more diverse pattern than that of the *C*. *jejuni* strains. Second, the heatmap can help detect consensus pattern of some DR/spacer across the input strains. For example, Fig. 7C illustrates that two DR sequences (inner number = ‘1’ and ‘6’) represented consensus patterns across the *C. jejuni* strains. Then we traced back to their IDs and sequences using CrisprVi, which resulted in ‘1’ for ‘DR_1433807’ etc.

(‘TTTTAGTCCCTTTTTAAATTTCTTTATGGTAAAATA’)

and ‘6’ for ‘DR_1533204’ etc.

(‘GTTTTAGTCCCTTTTTAAATTTCTTTATGGTAAAAT’).

Furthermore, we used the two sequences to query the local or online BLAST databases to validate the discovery. The blastn results showed that both of the sequences are *C. jejuni* specific without being found in *C. coli* or other species, which would be valuable for further study.

### Comparison of CRISPR visualization methods

**Fig. 8:**
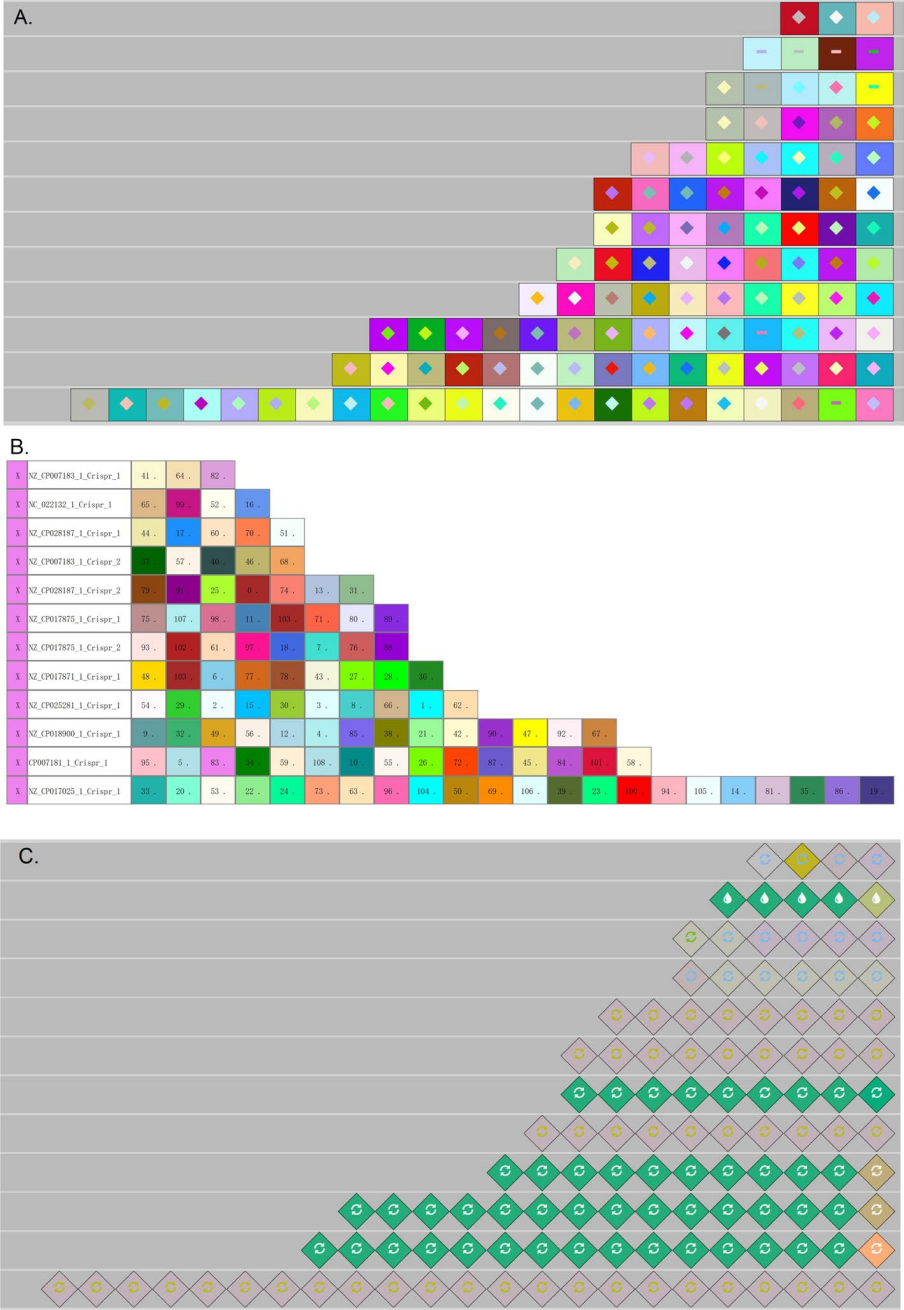

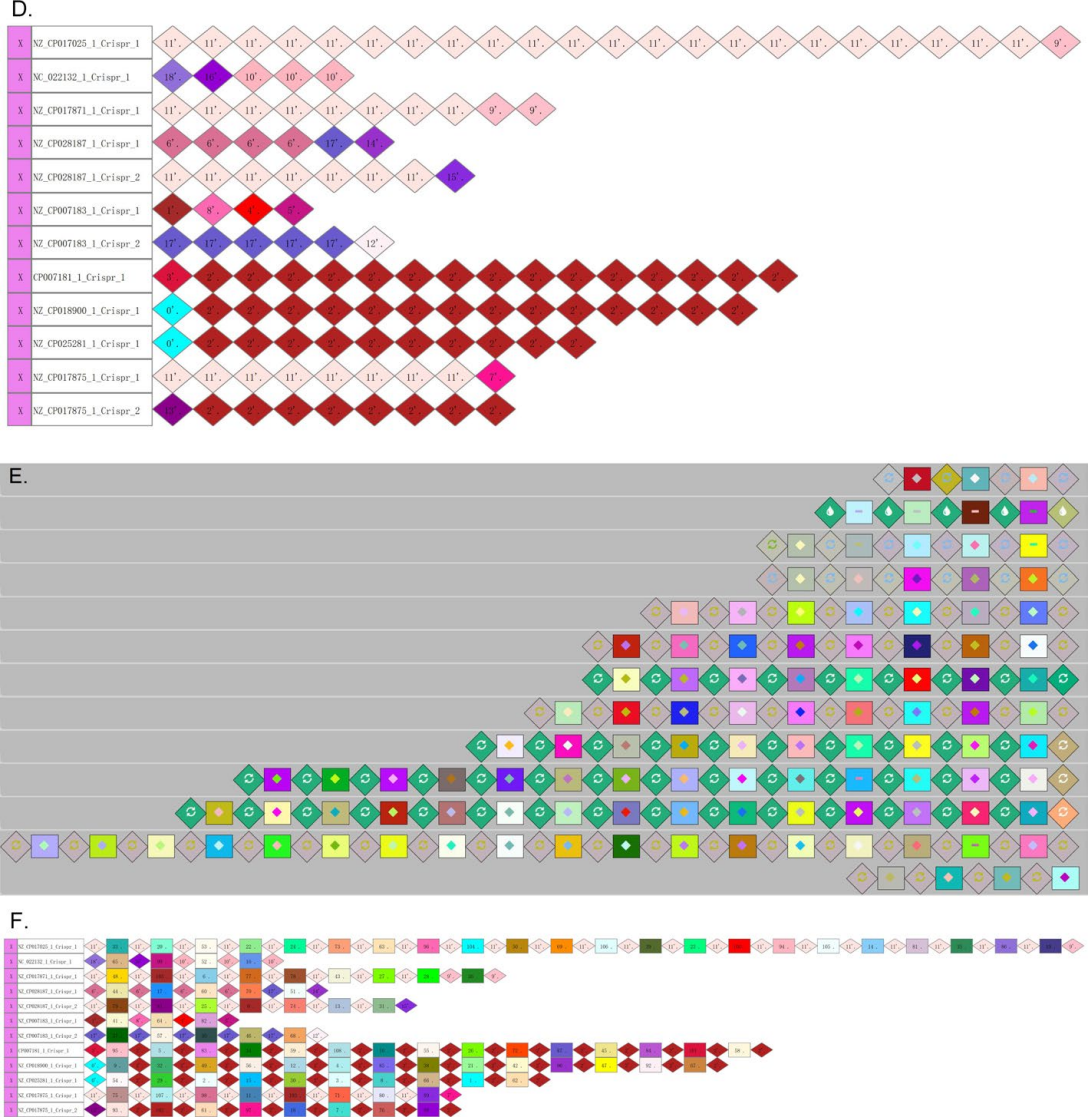

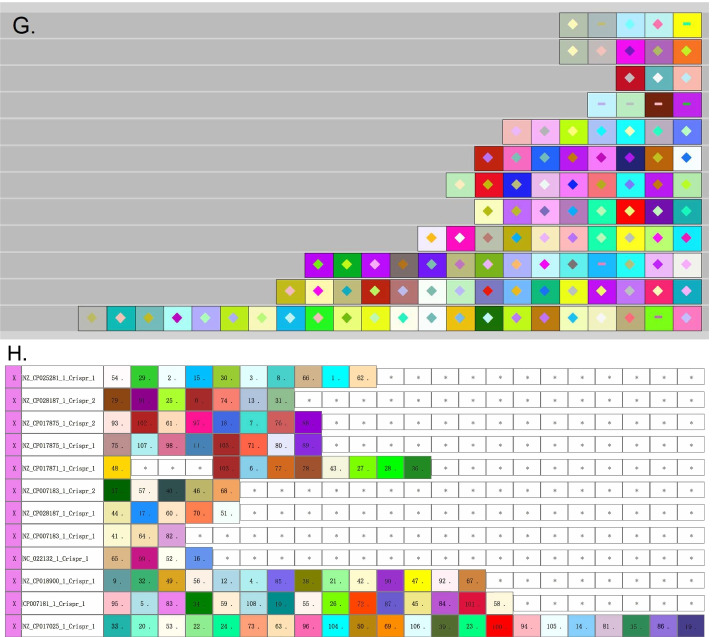
Comparison between CRISPRviz and CrisprVi visualization. **A** Sorting spacer arrays in CRISPRviz. **B** Sorting spacer arrays in CrisprVi. **C** Showing DRs in CRISPRviz. **D** Showing DRs in CrisprVi. **E** Showing DRs and spacers in CRISPRviz. **F** Showing DRs and spacers in CrisprVi. **G** Alignment of spacer arrays in CRISPRviz. **H** Alignment of spacer arrays in CrisprVi. In this figure, the visualized CRISPRs of the *C. coli* strains were predicted by MinCED

**Fig. 9:**
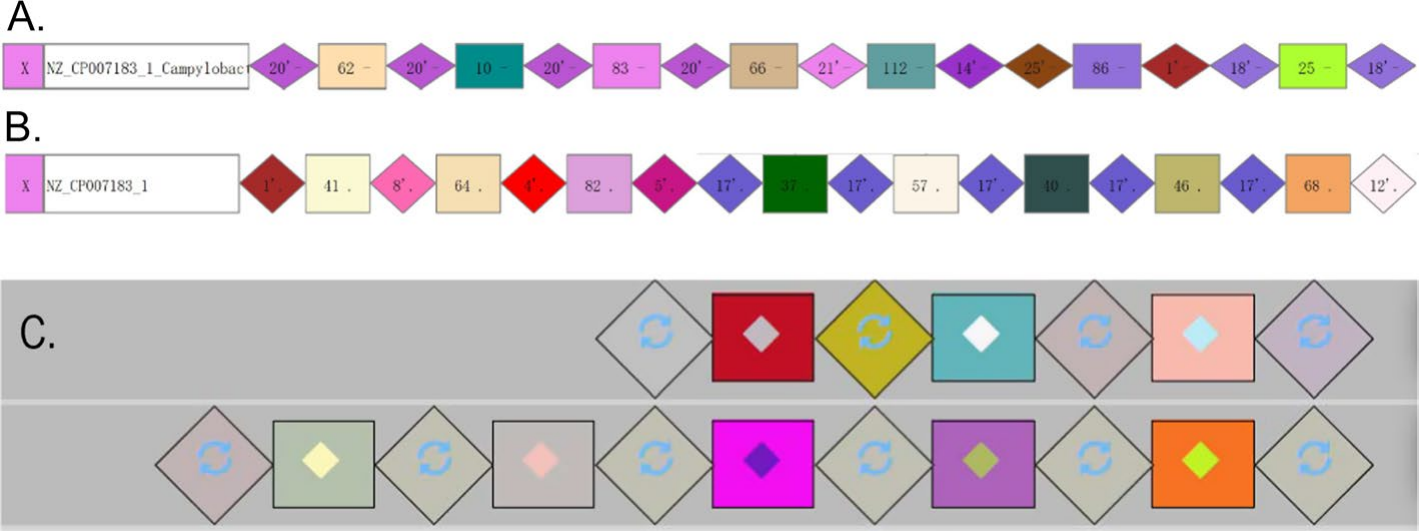
Differences of CRISPR visualization based on CRISPRCasFinder and MinCED. **A** CRISPRs of NZ_CP007183_1 (CRISPRCasFinder + CrisprVi). NZ_CP007183_1 is the ID of a *C. coli* strain. CRISPRCasFinder was used for CRISPR detection and CrisprVi was used for visualization. **B** CRISPRs of NZ_CP007183_1 (MinCED + CrisprVi). MinCED was used for CRISPR detection and CrisprVi was used for visualization. **C** CRISPRs of NZ_CP007183_1 (MinCED + CRISPRviz). MinCED was used for CRISPR detection and CRISPRviz was used for visualization

Several CRISPR visualization methods, e.g. CRISPRviz and CRISPRStudio, integrate CRISPR finding algorithms, which makes it difficult to compare these tools with our CrisprVi directly since current CrisprVi version does not contain the module for CRISPR finding. Since CRISPRStudio cannot visualize DR arrays, here we conducted comparison experiments between CrisprVi and CRISPRviz on dataset-I and dataset-II. As seen from Figs. 8 and 9, CrisprVi and CRISPRviz can both represent DRs and spacers in several ways, but they differ in some aspects.

#### CrisprVi and CRISPRviz show different color-symbol systems and operation

CrisprVi and CRISPRviz use different color-symbol systems and operation. Specifically, CRISPRviz displays the DRs and spacers in random combinations of colors and symbols, whereas in CrisprVi the colors of DR/spacer graphics are automatically assigned and can be changed manually (Fig. 3A). In addition, CrisprVi also uses inner number plus strand to denote one type of DRs/spacers uniquely (Fig. 3B). The color-symbol system of CRISPRviz or CrisprVi would become more and more complex as the number of DRs/spacers increases while CrisprVi can highlight the identical DRs/spacers by red boarders (Fig. 3C), which can help the users view the DRs/spacers of interest more clearly. Furthermore, the inner number used by CrisprVi are more useful for tracing back the meta data of the CRISPRs in such complex scenario.

#### CrisprVi accepts output from different CRISPR finding methods

Different CRISPR finding methods, e.g. CRISPRCasFinder [12] and MinCED [16], may give different prediction results. Once the users choose CRISPRviz for CRISPR visualization, they must acquiesce in the prediction accuracy of MinCED called by CRISPRviz. In comparison, CrisprVi does not connect to any specific CRISPR finding method, but the input files should follow the GFF format defined by CrisprVi or CRISPRCasFinder (see documentation of CrisprVi). Thus, the output GFF files of CRISPRCasFinder can be directly loaded to CrisprVi, while the CRISPR annotation files output by CRISPRviz/MinCED should be converted to our GFF format using the script *minced2gff.py* packaged in CrisprVi. Such conversion is important for the visualization comparison between CRISPRviz and CrisprVi as we can see the differences of CRISPR annotation in a visual way. As seen from Fig. 9, CrisprVi can arrange the two CRISPR arrays of a *C. coli* strain (NZ_CP007183_1) in the same track (Fig. 9A and B) while CRISPRviz only shows the two CRISPRs in two different tracks (Fig. 9C). The benefit of showing all CRISPRs of a strain in a line in CrisprVi is that the users can see the relative positions of the CRISPRs, and the boundary of two CRISPRs is just at the point of two connected DRs (Fig. 9A and B). Optionally, the users can arrange different CRISPR arrays of a strain in different tracks in CrisprVi as in CRISPRviz by selecting the CRISPR button on the board. Obviously, CRISPRCasFinder and MinCED gave different CRISPR prediction results on the same strain (e.g. NZ_CP007183_1). Moreover, CRISPRviz cannot show the array orientation, which is ignored by MinCED. Thus, the CrisprVi visualization based on MinCED prediction uses dot (‘.’) to indicate such missing information. But CRISPRviz does not present such comprehensive information of the CRISPR arrays as CrisprVi.

#### Differences in visualizing DRs/spacers

**Fig. 10:**
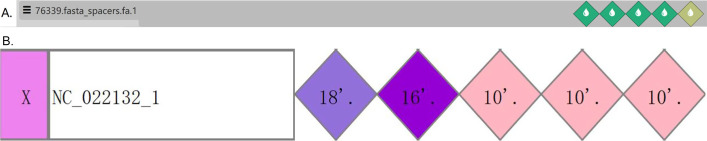
Comparison between CRISPviz and CrisprVi visualization of a DR array. **A** CRISPRviz visualization of a DR array. **B** CrisprVi visualization of a DR array. In this figure, the visualized CRISPRs of a *C. coli* strain NC_022132.1 was predicted by MinCED

In terms of visualizing the DRs/spacers, CRISPRviz and CrisprVi have different operation. For example, Fig. 10 illustrates a DR array composed of five repeats in the strain with ID ‘NC_022132.1’ (see Table 3 for details). As seen from Table 3, the DR array just has three types of sequences with slight differences, two of which are represented by the same color-symbol in CRISPRviz (Fig. 10A). Although the DRs with the same color-symbol (the green graphics in Fig. 10A) implies that they are similar to each other, their differences has been hidden (the sequences of No. 2 to 5 in Table 3), especially when the DR array have great diversity of DRs. Unfortunately, in such scenario the users cannot find any functions in CRISPRviz to inspect the differences between the similar DRs, which can only be dug out from the original annotation file. In comparison, CrisprVi assigns different color-number combinations to the DRs (Fig. 10B), which makes it easy to distinguish between the different DRs. In CrisprVi, the nucleotide composition of specific DR can be showed on the panel.

**Table 3:** Information of a DR array on ‘NC_022132.1’ genome

No	Start position	Sequence	Length
1	1463293	AACCGATTCTCTTTTTAAATTTCTTTATGGTAAAATA	37
2	1463359	GTTTTAGTCTCTTTTTAAATTTCTTTATGGTAAAATG	37
3	1463425	GTTTTAGTCTCTTTTTAAATTTCTTTATGGTAAAATA	37
4	1463491	GTTTTAGTCTCTTTTTAAATTTCTTTATGGTAAAATA	37
5	1463557	GTTTTAGTCTCTTTTTAAATTTCTTTATGGTAAAATA	37

#### Visualizing large datasets

Both CrisprVi and CRISPRviz can be used to visualize large datasets, which was validated on dataset-II containing 80 available strains with CRISPRs annotated by MinCED (see Additional files 3 and 4). Meanwhile, the visualization differences between the two tools still exist in such scenario.

#### Running time comparison

In terms of running time, CrisprVi and CRISPRviz include different components of running time. Specifically, CRISPRviz mainly includes spending time for DR/Spacer detection and data loading via web service while our CrisprVi include the time for data preprocessing, data loading locally, statistical analysis, and consensus sequencing finding. Thus, we only compared CrisprVi and CRISPRviz in terms of data loading via local network (see Table 4) on a laptop (Win10 64bit, Intel Core i5 CPU, 8 GB RAM). Meanwhile, CRISPRviz ran CRISPR detection on the same laptop with virtual Linux OS (Ubuntu 18.04). Five sub-datasets with varying size (20, 40, 60, 80 and 100) were generated by sampling from the original 100 strains of dataset-II. The CRISPRviz was run on each sub-dataset for visualization. Meanwhile, the CRISPR annotation files generated by MinCED/CRISPRviz were converted to GFF files, which were input to CrisprVi for visualization. The results manifest that CRISPRviz spent a lot of time for CRISPR detection and its data loading was much faster than our CrisprVi. Since CrisprVi integrates several types of functional objects for modelling, manipulating and analyzing the CRISPRs, and the number of the objects as well as the time for data loading increase as the sample size. The results indicate that the underlying efficiency of our visualization framework based on PyQt5 should be improved in the future.

**Table 4:** Running time comparison between CRISPRviz and CrisprVi

Sample size/count	Original	20	40	60	80	100
	After MinCED	14	34	52	63	80
CRISPRviz running time (s)	CRISPR detection	30	55	95	136	176
	data loading	0.6	0.7	0.8	0.9	1
CrisprVi running time (s)	data loading	6	10	24	42	58

#### Summary of CrisprVi, CRISPRviz and CRISPRStudio visualization

In compared with CrisprVi and CRISPRviz, CRISPRStudio just implements visualization of the spacers. Table 5 summarizes the similarities and differences of the main functions between them in terms of input files, DRs and spacers extraction methods, color-symbol system, alignment of spacer arrays, guide tree, statistics, etc.

**Table 5:** Comparison of main functions between CRISPRviz, CRISPRStudio and CrisprVi

No	Main functions	CRISPRviz	CRISPRStudio	CrisprVi
1	Input files	FASTA	FASTA	GFF, FASTA
2	Detect CRISPRs (DRs/spacers)	MinCED	CRISPRDetect	×
3	Color-symbol system	Glyphicon + color	Diamond + color	Number + strand + color
4	Change color	×	×	√
5	Pairwise alignment of spacers	Needleman-Wunsch algorithm	fasta36 [40]	Modified pairwise alignment
6	Guide tree	UPGMA	UPGMA	UPGMA
7	Show DRs and spacers	√	×	√
8	Show repeats only	√	√	√
9	Show spacers only	√	×	√
10	Align spacers	√	√	√
11	Sort by length	√	×	√
12	Highlight all identical sequences	×	√	√
13	Show information of specific graphics	×	×	√
14	Statistics of DRs/spacers	×	×	√
15	Consensus DR/spacer sequences finding	×	×	√

‘×’ means that the corresponding tool does not have the function

‘√’ means that the corresponding tool has the function

## Discussion

Since the role of CRISPR-Cas system was revealed a decade ago, a diversity of CRISPR-Cas systems with various physiological functions have been discovered in prokaryotes. Currently the CRISPR-Cas systems can be categorized into two classes with six types and additional subtypes, and new types might remain to be discovered [38]. From an evolutionary view, it is of importance to investigate the CRISPR distribution across strains of interest. Fortunately, computational tools such as CRISPRviz and CRISPRStudio have provided researchers with a large scale analysis to find and visualize the CRISPR arrays [39]. However, the functions of current tools are not complete enough and few tools are available for CRISPR visualization. Therefore, novel tools for CRISPR analysis are still in demand.

To provide an efficient CRISPR visualization tool to the community, here we proposed a Python package named CrisprVi. By the GUI of CrisprVi, the information of CRISPR sequences including DRs and spacers can be visually displayed, and the users can also manipulate the CRISPR arrays on the screen. In comparison to other tools, CrisprVi not only improves functions and effects for CRISPR visualization, but also helps conduct several statistical analysis of the CRISPR sequences, and show the results in different ways. In addition, CrisprVi provides a method for analysing consensus sequences of DRs/spacers based on BLAST and clustering. Overall, CrisprVi can be used to compare and analyze the CRISPR information of multiple strains intuitively. In the experiment, CrisprVi was successfully tested on different magnitude of datasets and scenarios, which also indicates that our tool can be used for analysing other prokaryotic CRISPR strains. Although CrisprVi does not provide the function of CRISPR detection, it could be more flexible than those one-step processing tools that hides CRISPR detection, as the users can choose any available CRISPR finding tool and conduct curation on the automatically predicted results before CrisprVi visualization. In the future, CrisprVi is planned to be integrated with methods for complete CRISPR-Cas system identification and strain typing, including CRISPR finding, cas genes detection, etc.

## Conclusions

CrisprVi is a convenient tool for visualizing and analyzing the CRISPR sequences and it would be helpful for researchers to inspect novel CRISPR-Cas systems of prokaryotes.

### Availability and requirements

- Project name: CrisprVi
- Project home page: https://sourceforge.net/projects/crisprvi
- Operating system(s): Platform independent
- Programming language: Python
- Other requirements: Python 3.7, PyQt5, Numpy, pandas, matplotlib, seaborn, Biopython, BLAST 2.9.0
- License: GNU GPL (version 3)
- Any restrictions to use by non-academics: No

## Supplementary Information

**Additional file 1. Table S1:** Summary of dataset-I. Dataset-I includes core genomes of 12 *Campylobacter coli* (*C. coli*) and 12 *Campylobacter jejuni* (*C. jejuni*) strains.

**Additional file 2. Table S2:** Summary of dataset-II. Dataset-II includes DNA sequences of 100 prokaryotic strains.

**Additional file 3. Fig. S1:** Snapshot of visualizing CRISPRs of 80 strains on CrisprVi.

**Additional file 4. Fig. S2:** Snapshot of visualizing CRISPRs of 80 strains on CRISPRviz.

## Data Availability

The datasets analyzed during the current study are available at: https://sourceforge.net/projects/crisprvi/files/data/dataset-1.zip and https://sourceforge.net/projects/crisprvi/files/data/dataset-2.zip.

## Abbreviations

CRISPR: Clustered regularly interspaced short palindromic repeats
Cas: CRISPR-associated
ID: Identifier
GUI: Graphic user interface
BLAST: Basic Local Alignment Search Tool
GFF: General feature format
DR: Direct repeat
*C. coli*: *Campylobacter coli*
*C. jejuni*: *Campylobacter jejuni*
