# Supplementary material for: CrisprVi: a software for visualizing and analyzing CRISPR sequences of prokaryotes
Source: BMC Bioinformatics. 2022 May 11;23(Suppl 3):172. doi: 10.1186/s12859-022-04716-9 (PMC9128103; doi:10.1186/s12859-022-04716-9)
Supplement: Supplementary file 1 — Additional file 1. Table S1: Summary of dataset-I. Dataset-I includes core genomes of 12 Campylobacter coli (C. coli) and 12 Campylobacter jejuni (C. jejuni) strains. [file 12859_2022_4716_MOESM1_ESM.pdf]

Table S1 Summary of dataset-I

Source	Strain ID (accession number)	References
Campylobacter_coli_RM4661, complete genome	CP007181.1	[1]
Campylobacter_coli_76339, complete sequence	NC_022132.1	[2]
Campylobacter_coli_RM1875	NZ_CP007183.1	[3]
Campylobacter_coli_strain_CF2-75	NZ_CP013036.1	[4]
Campylobacter_coli_strain_14983A	NZ_CP017025.1	[5]
Campylobacter_coli_strain_YF2105	NZ_CP017865.1	[6]
Campylobacter_coli_strain_BP3183	NZ_CP017871.1	[7]
Campylobacter_coli_strain_WA333	NZ_CP017873.1	[8]
Campylobacter_coli_strain_ZV1224	NZ_CP017875.1	[9]
Campylobacter_coli_strain_YH502	NZ_CP018900.1	[10]
Campylobacter_coli_strain_YH503	NZ_CP025281.1	[11]
Campylobacter_coli_strain_CFSAN054106	NZ_CP028187.1	[12]
Campylobacter_jejuni_subsp__jejuni_NCTC_11168-BN148, complete sequence	NC_018521.1	[13]
Campylobacter_jejuni_subsp__jejuni_00-2538, complete sequence	NC_022351.2	[14]
Campylobacter_jejuni_subsp__jejuni_00-2426, complete sequence	NC_022352.2	[14]
Campylobacter_jejuni_subsp__jejuni_00-2544, complete sequence	NC_022353.2	[14]
Campylobacter_jejuni_subsp__jejuni_00-2425, complete sequence	NC_022362.2	[14]
Campylobacter_jejuni_4031, complete sequence	NC_022529.1	[15]
Campylobacter_jejuni_subsp__jejuni_NCTC_11168-GSv	NZ_CP006689.1	[16]
Campylobacter_jejuni_subsp__jejuni_strain_01-1512	NZ_CP010072.1	[17]
Campylobacter_jejuni_subsp__jejuni_strain_00-1597	NZ_CP010306.1	[18]
Campylobacter_jejuni_subsp__jejuni_strain_00-6200	NZ_CP010307.1	[19]
Campylobacter_jejuni_strain_11168H_lacY	NZ_CP022439.1	[20]
Campylobacter_jejuni_strain_81-176_G1_B0	NZ_CP022440.1	[21]

1. **Campylobacter coli RM4661 chromosome, complete genome** [https://www.ncbi.nlm.nih.gov/nuccore/CP007181.1/]

2. Skarp-de Haan CP, Culebro A, Schott T, Revez J, Schweda EK, Hänninen ML, Rossi M: **Comparative genomics of unintrogressed Campylobacter coli clades 2 and 3**. *BMC Genomics* 2014, **15**:129.
3. **Campylobacter coli RM1875 chromosome, complete genome** [https://www.ncbi.nlm.nih.gov/nuccore/NZ_CP007183.1]
4. **Campylobacter coli strain CF2-75 genome** [https://www.ncbi.nlm.nih.gov/nuccore/NZ_CP013036.1]
5. **Campylobacter coli strain 14983A, complete sequence** [https://www.ncbi.nlm.nih.gov/nuccore/NZ_CP017025.1]
6. **Campylobacter coli strain YF2105 chromosome, complete genome** [https://www.ncbi.nlm.nih.gov/nuccore/NZ_CP017865.1]
7. **Campylobacter coli strain BP3183 chromosome, complete genome** [https://www.ncbi.nlm.nih.gov/nuccore/NZ_CP017871.1]
8. **Campylobacter coli strain WA333 chromosome, complete genome** [https://www.ncbi.nlm.nih.gov/nuccore/NZ_CP017873.1]
9. **Campylobacter coli strain ZV1224 chromosome, complete genome** [https://www.ncbi.nlm.nih.gov/nuccore/NZ_CP017875.1]
10. **Campylobacter coli strain YH502 chromosome, complete genome** [https://www.ncbi.nlm.nih.gov/nuccore/NZ_CP018900.1]
11. **Campylobacter coli strain YH503 chromosome, complete genome** [https://www.ncbi.nlm.nih.gov/nuccore/NZ_CP025281.1]
12. **Campylobacter coli strain CFSAN054106 chromosome, complete genome** [https://www.ncbi.nlm.nih.gov/nuccore/NZ_CP028187.1]
13. Revez J, Schott T, Rossi M, Hänninen ML: **Complete genome sequence of a variant of Campylobacter jejuni NCTC 11168**. *J Bacteriol* 2012, **194**(22):6298-6299.
14. Clark CG, Chong PM, McCorrister SJ, Simon P, Walker M, Lee DM, Nguy K, Cheng K, Gilmour MW, Westmacott GR: **The CJIE1 prophage of Campylobacter jejuni affects protein expression in growth media with and without bile salts**. *BMC microbiology* 2014, **14**:70.
15. **Campylobacter jejuni 4031 chromosome I, complete sequence** [https://www.ncbi.nlm.nih.gov/nuccore/NC_022529.1]
16. Thomas DK, Lone AG, Selinger LB, Taboada EN, Uwiera RR, Abbott DW, Inglis GD: **Comparative variation within the genome of Campylobacter jejuni NCTC 11168 in human and murine hosts**. *PLoS One* 2014, **9**(2):e88229.
17. **Campylobacter jejuni subsp. jejuni strain 01-1512 chromosome, complete genome** [https://www.ncbi.nlm.nih.gov/nuccore/NZ_CP010072.1]
18. **Campylobacter jejuni subsp. jejuni strain 00-1597 chromosome, complete genome** [https://www.ncbi.nlm.nih.gov/nuccore/NZ_CP010306.1]
19. **Campylobacter jejuni subsp. jejuni strain 00-6200 chromosome, complete genome** [https://www.ncbi.nlm.nih.gov/nuccore/NZ_CP010307.1]
20. **Campylobacter jejuni strain 11168H/lacY chromosome, complete genome** [https://www.ncbi.nlm.nih.gov/nuccore/NZ_CP022439.1]

21. **Campylobacter jejuni strain 81-176_G1_B0 chromosome, complete genome** [https://www.ncbi.nlm.nih.gov/nuccore/NZ_CP022440.1]
