# Supplementary material for: CrisprVi: a software for visualizing and analyzing CRISPR sequences of prokaryotes
Source: BMC Bioinformatics. 2022 May 11;23(Suppl 3):172. doi: 10.1186/s12859-022-04716-9 (PMC9128103; doi:10.1186/s12859-022-04716-9)
Supplement: Supplementary file 2 — Additional file 2. Table S2: Summary of dataset-II. Dataset-II includes DNA sequences of 100 prokaryotic strains. [file 12859_2022_4716_MOESM2_ESM.pdf]

Table S2 Summary of dataset-II

Source	Strain ID (accession number)	References
Amedibacterium intestinale JCM 30884 DNA, complete genome	AP019695.1	[1]
Acaryochloris marina MBIC11017, complete genome	CP000828.1	[2]
Acetobacter aceti JCM20276 DNA, complete genome	AP023326.1	[3]
Acetobacter aceti NBRC 14818 DNA, complete genome	AP023410.1	[4]
Acetobacter pasteurianus subsp. paradoxus strain LMG 1591 plasmid unnamed1, complete sequence	CP015169.1	[5]
Acetobacter orientalis FAN1 DNA, complete genome	AP018515.1	[6]
Acetobacter oryzifermentans strain dm chromosome, complete genome	CP022374.1	[7]
Acetobacter sp. SLV-7, complete genome	CP011120.1	[8]
Acetobacter oryzoeni strain B6 chromosome, complete genome	CP042808.1	[9]
Acetobacter pasteurianus strain CICC 22518 plasmid pAP22518-1, complete sequence	CP039845.1	[10]
Acetobacter pasteurianus IFO 3283-01 plasmid pAPA01-011 DNA, complete genome	AP011122.1	[11]
Acetobacter pomorum strain BDGP5 chromosome, complete genome	CP023657.1	[12]
Acetobacter sp. JWB chromosome, complete genome	CP030871.1	[13]
Acetobacter tropicalis strain BDGP1 chromosome, complete genome	CP022699.1	[14]
Complete chromosome Acholeplasma palmae	FO681347.1	[15]
Achromobacter denitrificans strain FDAARGOS_786 chromosome, complete genome	CP053987.1	[16]
Achromobacter sp. AONIH1 chromosome, complete genome	CP026124.1	[17]
Acidaminococcus fermentans DSM 20731, complete genome	CP001859.1	[18]

Acidianus ambivalens strain LEI 10 chromosome, complete genome	CP045482.1	[19]
Acidiphilium cryptum JF-5 plasmid pACRY02, complete sequence	CP000690.1	[20]
Acidipropionibacterium virtanenii strain JS278 chromosome, complete genome	CP025198.1	[21]
Acidithiobacillus caldus ATCC 51756, complete genome	CP005986.1	[22]
Acidobacteria bacterium Mor1 sequence	CP011806.1	[23]
Acidovorax avenae subsp. avenae strain INDB2 chromosome	CP028292.1	[24]
Acidovorax cattleyae strain CAT98_1 chromosome	CP028290.1	[24]
Acidovorax sp. JS42, complete genome	CP000539.1	[25]
Aciduliprofundum boonei T469, complete genome	CP001941.1	[26]
Acinetobacter baumannii genome assembly R2091, chromosome: I	LN997846.1	[27]
Acinetobacter baumannii strain 11W359501 chromosome	CP041035.1	[28]
Acinetobacter sp. ADP1 complete genome	CR543861.1	[29]
Acinetobacter bereziniae strain GD0320 chromosome, complete genome	CP066121.1	[30]
Acinetobacter chinensis strain WCHAc010005 chromosome, complete genome	CP032134.1	[31]
Acinetobacter guillouiae DNA, complete geonome, strain: NBRC 110550	AP014630.1	[32]
Acinetobacter haemolyticus strain 2126ch chromosome, complete genome	CP031991.1	[33]
Acinetobacter indicus strain FS42-2 chromosome, complete genome	CP046595.1	[34]
Acinetobacter johnsonii strain E10B chromosome, complete genome	CP059080.1	[35]
Acinetobacter larvae strain BRTC-1 chromosome, complete genome	CP016895.1	[36]
Acinetobacter piscicola strain YH12207_T chromosome, complete genome	CP048659.1	[37]
Acinetobacter radioresistens strain DD78 chromosome, complete genome	CP038022.1	[38]
Acinetobacter seifertii strain AS11 chromosome, complete genome	CP061678.1	[39]

Acinetobacter soli strain GFJ2, complete genome	CP016896.1	[40]
Acinetobacter sp. 10FS3-1 chromosome, complete genome	CP039143.1	[41]
Acinetobacter ursingii M3 DNA, chromosome 1, complete genome	AP018824.1	[42]
Acinetobacter venetianus strain TUST-DM21 chromosome, complete genome	CP060161.1	[43]
Acinetobacter wuhouensis strain WCHA60 chromosome, complete genome	CP031716.1	[44]
Actinobacillus equuli strain NCTC9435 genome assembly, chromosome: 1	LR134486.1	[45]
Actinobacillus indolicus strain AIFJ1607 chromosome, complete genome	CP038145.1	[46]
Actinobacillus lignieresii strain NCTC10568 genome assembly, chromosome: 1	LR134169.1	[47]
Actinobacillus pleuropneumoniae strain KL 16 chromosome, complete genome	CP022715.1	[48]
Actinomyces gaoshouyii strain pika_114 chromosome, complete genome	CP020468.1	[49]
Actinomyces israelii strain NCTC12972 genome assembly, chromosome: 1	LR134357.1	[50]
Actinomyces naeslundii DNA, complete genome	AP017894.1	[51]
Actinomyces oris strain FDAARGOS_1051 chromosome, complete genome	CP066060.1	[52]
Actinomyces sp. Marseille-P2985 strain Marseille-P2985T genome assembly, chromosome: contig00001	LT635457.1	[53]
Actinomyces sp. Chiba101 DNA, complete genome	AP017896.1	[54]
Actinotignum schaalii strain CCUG 27420 chromosome, complete genome	CP008802.1	[55]
Adhaeribacter swui strain KCTC 52873 chromosome, complete genome	CP055156.1	[56]
Advenella mimigardefordensis DPN7, complete genome	CP003915.1	[57]
Aerococcaceae bacterium zg-1292 chromosome, complete genome	CP065955.1	[58]

Aerococcus christensenii strain CCUG28831, complete genome	CP014159.1	[59]
Aerococcus sanguinicola strain CCUG43001, complete genome	CP014160.1	[59]
Aerococcus urinae strain CCUG36881, complete genome	CP014161.1	[59]
Aerococcus viridans strain FDAARGOS_672 chromosome, complete genome	CP046334.1	[60]
Aeromicrobium choanae strain 9H-4 genome assembly, chromosome: 1	LT796768.1	[61]
Aeromonas caviae strain 1607-10029 chromosome, complete genome	CP047981.1	[62]
Aeromonas media strain MC64 plasmid pMC64A, complete sequence	CP047963.1	[63]
Aeromonas simiae strain A6 chromosome, complete genome	CP040449.1	[64]
Aeromonas veronii strain 17ISAe chromosome, complete genome	CP028133.1	[65]
Aeropyrum camini SY1 = JCM 12091 DNA, complete genome	AP012489.1	[66]
Aeropyrum pernix K1 DNA, complete genome	BA000002.3	[67]
Aerosticca soli DNA, complete genome	AP018560.1	[68]
Afipia sp. GAS231 genome assembly, chromosome: I	LT629703.1	[69]
Agarilytica rhodophyticola strain 017 chromosome, complete genome	CP020038.1	[70]
Agarivorans gilvus strain WH0801, complete genome	CP013021.1	[71]
Aggregatibacter actinomycetemcomitans strain 624, complete genome	CP012959.1	[72]
Aggregatibacter aphrophilus strain NCTC5906 genome assembly, chromosome: I	LR134327.1	[73]
Aggregatibacter segnis strain FDAARGOS_987 chromosome, complete genome	CP065983.1	[52]
Agrobacterium rhizogenes strain K599 chromosome 1, complete genome	CP019701.2	[74]

Agrobacterium tumefaciens strain CFBP6625 chromosome circular, complete genome	CP039910.1	[75]
Agrococcus sp. SGAir0287 chromosome, complete genome	CP027942.1	[76]
Agromyces aureus strain AR33, complete genome	CP013979.1	[77]
Akkermansia glycaniphila isolate APytT genome assembly, chromosome: I	LT629973.1	[78]
Akkermansia muciniphila isolate MGYG-HGUT-02454 genome assembly, chromosome: I	LR699001.1	[79]
Alcaligenaceae bacterium SJ-26 chromosome, complete genome	CP041732.1	[80]
Alcaligenes aquatilis strain BU33N genome	CP022390.1	[81]
Alcaligenes aquatilis strain BU33N genome, complete genome	CP032521.1	[82]
Alcanivorax dieselolei B5, complete genome	CP003466.1	[83]
Alcanivorax pacificus W11-5, complete genome	CP004387.1	[84]
Alcanivorax xenomutans strain P40, complete genome	CP012331.1	[85]
Algibacter alginicilyticus strain HZ22, complete genome	CP012898.1	[86]
Algoriphagus sp. Y33 chromosome, complete genome	CP061947.1	[86]
Alicycliphilus denitrificans strain DP3 chromosome, complete genome	CP051298.1	[87]
Alicyclobacillus acidocaldarius subsp. acidocaldarius DSM 446, complete genome	CP001727.1	[87]
Alienimonas californiensis strain CA12 chromosome	CP036265.1	[88]
Aliarcobacter faecis strain CCUG 66484 chromosome, complete genome	CP053837.1	[89]
Aliivibrio wodanis genome assembly AWOD1, chromosome : 2	LN554847.1	[90]
Alistipes sp. Marseille-P5997 genome assembly, chromosome: contig00001	LR027382.1	[90]
Alistipes onderdonkii subsp. vulgaris 3BBH6 DNA, complete genome	AP019734.1	[91]

Allochromatium vinosum DSM 180 plasmid pALVIN01, complete sequence	CP001897.1	[92]
Allokutzneria albata strain DSM 44149 genome assembly, chromosome: I	LT629701.1	[93]

1. Ikeyama N, Toyoda A, Morohoshi S, Kunihiro T, Murakami T, Mori H, et al. Amedibacterium intestinale gen. nov., sp. nov., isolated from human faeces, and reclassification of Eubacterium dolichum Moore et al. 1976 (Approved Lists 1980) as Amedibacillus dolichus gen. nov., comb. nov. 2019. https://www.ncbi.nlm.nih.gov/nuccore/AP019695.1. Accessed 20 Jun 2021.
2. Swingley WD, Chen M, Cheung PC, Conrad AL, Dejesa LC, Hao J, et al. Niche adaptation and genome expansion in the chlorophyll d-producing cyanobacterium Acaryochloris marina. 2007. https://www.ncbi.nlm.nih.gov/nuccore/CP000828.1. Accessed 20 Jun 2021.
3. Hirose Y, Mihara H. Complete Genome Sequence of an acetic acid bacterium, Acetobacter aceti JCM20276. 2020. https://www.ncbi.nlm.nih.gov/nuccore/AP023326.1. Accessed 20 Jun 2021.
4. Sakurai K, Arai H, Ishii M, Igarashi Y. Transcriptome response to different carbon sources in Acetobacter aceti. Microbiology (Reading). 2011;157(Pt 3):899-910.
5. Jeon CO, Cho GY, Jeong HI, Kim KH. Acetobacter pasteurianus subsp. paradoxus strain LMG 1591 plasmid unnamed1, complete sequence. 2016. https://www.ncbi.nlm.nih.gov/nuccore/CP015169.1. Accessed 20 Jun 2021.
6. Nakashima N, Tamura T. Acetobacter orientalis genome. 2018. https://www.ncbi.nlm.nih.gov/nuccore/AP018515.1. Accessed 20 Jun 2021.
7. Simhadri RK, Guo R, Fast EM, Schultz MJ, Vaisman N, Slatko B, et al. The gut commensal microbiome of Drosophila is modified by the endosymbiont Wolbachia. 2017. https://www.ncbi.nlm.nih.gov/nuccore/CP022374.1. Accessed 20 Jun 2021.
8. Cho GY, Jeon CO. Genome study of Acetobacter sp. SLV-7. 2015. https://www.ncbi.nlm.nih.gov/nuccore/CP011120.1. Accessed 20 Jun 2021.
9. Baek JH, Kim KH, Jeon CO, Han DM. Acetobacter oryzioeni sp. nov., isolated from Korean rice wine vinegar. 2019. https://www.ncbi.nlm.nih.gov/nuccore/CP042808.1. Accessed 20 Jun 2021.
10. Zhao J-Y, Xiao Z. Genome sequences of Acetobacter pasteurianus CICC 22518. 2019. https://www.ncbi.nlm.nih.gov/nuccore/CP039845.1. Accessed 20 Jun 2021.
11. Azuma Y, Hosoyama A, Matsutani M, Furuya N, Horikawa H, Harada T, et al. Whole-genome analyses reveal genetic instability of Acetobacter pasteurianus. 2009. https://www.ncbi.nlm.nih.gov/nuccore/AP011122.1. Accessed 20 Jun 2021.
12. Wan KH, Yu C, Park S, Hammonds AS, Booth BW, Celniker SE. Complete Genome Sequence of Acetobacter pomorum Oregon-R-modENCODE STRAIN

BDGP5, an acetic acid bacterium isolated from Drosophila melanogaster gut. 2017. https://www.ncbi.nlm.nih.gov/nuccore/CP023657.1. Accessed 20 Jun 2021.
13. Bae J-W. Complete genome sequencing of Acetobacter sp. JWB. 2018. https://www.ncbi.nlm.nih.gov/nuccore/CP030871.1. Accessed 20 Jun 2021.
14. Wan KH, Yu,C., Park S, Hammonds AS, Booth BW, Celniker SE. Complete Genome Sequence of Acetobacter tropicalis Oregon-R-modENCODE STRAIN BDGP1, an acetic acid bacterium isolated from Drosophila melanogaster gut. 2017. https://www.ncbi.nlm.nih.gov/nuccore/CP022699.1. Accessed 20 Jun 2021.
15. Kube M, Siewert C, Migdoll AM, Duduk B, Holz S, Rabus R, et al. Analysis of the complete genomes of Acholeplasma brassicae, A. palmae and A. laidlawii and their comparison to the obligate parasites from 'Candidatus Phytoplasma'. J Mol Microbiol Biotechnol. 2014;24(1):19-36.
16. Sproer C, Gronow S, Severitt S, Schroder I, Tallon L, Sadzewicz L, et al. FDA dAtabase for Regulatory Grade micrObial Sequences (FDA-ARGOS): Supporting development and validation of Infectious Disease Dx tests. 2020. https://www.ncbi.nlm.nih.gov/nuccore/CP053987.1. Accessed 20 Jun 2021.
17. Weingarten RA, Johnson RC, Conlan S, Ramsburg AM, Dekker JP, Lau AF, et al. Genomic Analysis of Hospital Plumbing Reveals Diverse Reservoir of Bacterial Plasmids Conferring Carbapenem Resistance. 2018. https://www.ncbi.nlm.nih.gov/nuccore/CP026124.1. Accessed 20 Jun 2021.
18. Chang YJ, Pukall R, Saunders E, Lapidus A, Copeland A, Nolan M, et al. Complete genome sequence of Acidaminococcus fermentans type strain (VR4). 2010. https://www.ncbi.nlm.nih.gov/nuccore/CP001859.1. Accessed 20 Jun 2021.
19. Counts JA, Kelly RM. Genome Sequences from Six Type Strain Members of the Archaeal Family Sulfolobaceae: Acidianus ambivalens, Acidianus infernus, Metallosphaera prunae, Stygiolobus azoricus, Sulfolobus metallicus, and Sulfurisphaera ohwakuensis. 2019. https://www.ncbi.nlm.nih.gov/nuccore/CP045482.1. Accessed 20 Jun 2021.
20. Copeland A, Lucas S, Lapidus A, Barry K, Detter JC, Glavinadel Rio T, et al. Complete sequence of plasmid2 pACRY02 of Acidiphilium cryptum JF-5. 2007. https://www.ncbi.nlm.nih.gov/nuccore/CP000690.1. Accessed 20 Jun 2021.
21. Laine P, Deptula P, Varmanen P, Auvinen P. The whole genome sequence of the Acidipropionibacterium virtanenii sp. nov. type strain JS278. 2017. https://www.ncbi.nlm.nih.gov/nuccore/CP025198.1. Accessed 20 Jun 2021.
22. Valdes J, Quatrini R, Hallberg K, Dopson M, Valenzuela PD, Holmes DS. Draft genome sequence of the extremely acidophilic bacterium Acidithiobacillus caldus ATCC 51756 reveals metabolic versatility in the genus Acidithiobacillus. J Bacteriol. 2009;191(18):5877-8.
23. Cummings S, Barbe D, Ferreira Leao T, Korobeynikov A, Engene N, Glukhov E, et al. A Novel Uncultured Heterotrophic Bacterial Associate of the Cyanobacterium Moorea producens JHB. 2015. https://www.ncbi.nlm.nih.gov/nuccore/CP011806.1. Accessed 20 Jun 2021.
24. Zeng Q, Wang J, Bertels F, Giordano PR, Chilvers MI, Huntley RB, et al. Recombination of Virulence Genes in Divergent Acidovorax avenae Strains That

Infect a Common Host. 2018. https://www.ncbi.nlm.nih.gov/nuccore/CP028292.1. Accessed 20 Jun 2021.
25. Copeland A, Lucas S, Lapidus A, Barry K, Detter JC, Glavinadel Rio T, et al. Complete sequence of Chromosome1 of Acidovorax sp. JS42. 2006. https://www.ncbi.nlm.nih.gov/nuccore/CP000539.1. Accessed 20 Jun 2021.
26. Lucas S, Copeland A, Lapidus A, Cheng J-F, Bruce D, Goodwin L, et al. Complete sequence of Aciduliprofundum boonei T469. 2010. https://www.ncbi.nlm.nih.gov/nuccore/CP001941.1. Accessed 20 Jun 2021.
27. Wibberg, Daniel. Acinetobacter baumannii genome assembly R2091, chromosome : I. 2015. https://www.ncbi.nlm.nih.gov/nuccore/LN997846.1. Accessed 20 Jun 2021.
28. Lambert C, Connerton I, Negus D, Sockett L. Acinetobacter baumannii strain 11W359501 chromosome, complete genome. 2019. https://www.ncbi.nlm.nih.gov/nuccore/CP041035.1. Accessed 20 Jun 2021.
29. Barbe V, Vallenet D, Fonknechten N, Kreimeyer A, Oztas S, Labarre L, et al. Unique features revealed by the genome sequence of Acinetobacter sp. ADP1, a versatile and naturally transformation competent bacterium. 2004. https://www.ncbi.nlm.nih.gov/nuccore/CR543861.1. Accessed 20 Jun 2021.
30. Wong N-K, Pan Q. Characterization of Tn125 harboring carbapenem-resistant Acinetobacter bereziniae clinical isolates. 2020. https://www.ncbi.nlm.nih.gov/nuccore/CP066121.1. Accessed 20 Jun 2021.
31. Hu Y, Feng Y, Qin J, Zhang X, Zong Z. Acinetobacter chinensis, a novel Acinetobacter species, carrying blaNDM-1, recovered from hospital sewage. 2017. https://www.ncbi.nlm.nih.gov/nuccore/CP032134.1. Accessed 20 Jun 2021.
32. Yee L, Hosoyama A, Ohji S, Tsuchikane K, Shimodaira J, Yamazoe A, et al. Complete Genome Sequence of a Dimethyl Sulfide-Utilizing Bacterium, Acinetobacter guillouiae Strain 20B (NBRC 110550). Genome Announc. 2014;2(5).
33. Castro-Jaimes S, Cevallos MA. Analysis of the genomic diversity of Mexican Acinetobacter haemolyticus clinical isolates. 2018. https://www.ncbi.nlm.nih.gov/nuccore/CP031991.1. Accessed 20 Jun 2021.
34. Cui C, Chen C, Sun J, Liu Y. Non-baumannii Acinetobacter spp. carrying blaNDM-1 isolated in China. 2019. https://www.ncbi.nlm.nih.gov/nuccore/CP046595.1. Accessed 20 Jun 2021.
35. Ghaly TM, Sajjad A, Tetu SG, Gillings MR. A family of multi-drug resistance mega-plasmids in Acinetobacter species. 2020. https://www.ncbi.nlm.nih.gov/nuccore/CP059080.1. Accessed 20 Jun 2021.
36. Liu S. Acinetobacter larvae strain BRTC-1 chromosome, complete genome. 2016. https://www.ncbi.nlm.nih.gov/nuccore/CP016895.1. Accessed 20 Jun 2021.
37. Chen C, Sun J, Liao X-P, Liu Y-H. Tigecycline-resistant Acinetobacter species from pigs and migratory birds. 2020. https://www.ncbi.nlm.nih.gov/nuccore/CP048659.1. Accessed 20 Jun 2021.
38. Macaya C, Duran RE, Mendez V, Aguila P, Salva-Serra F, Jaen-Luchoro D, et al. Complete genome sequence of Acinetobacter radioresistens DD78. 2019. https://www.ncbi.nlm.nih.gov/nuccore/CP038022.1. Accessed 20 Jun 2021.

39. Li L-H, Yang Y-S, Sun J-R, Huang T-W, Huang W-C, Wang Y-C, et al. Clinical and molecular characterization of Acinetobacter seifertii in Taiwan. 2020. https://www.ncbi.nlm.nih.gov/nuccore/CP061678.1. Accessed 20 Jun 2021.
40. Tabata M, Kuboki S, Gibu N, Kinouchi Y, Vangnai A, Kasai D, et al. Complete genome sequence of Acinetobacter baylyi strain GFJ2. 2016. https://www.ncbi.nlm.nih.gov/nuccore/CP016896.1. Accessed 20 Jun 2021.
41. Chen C, Sun J, Liao X-P, Liu Y-H. Whole genome sequencing data of Acinetobacter sp. 10FS3-1. 2019. https://www.ncbi.nlm.nih.gov/nuccore/CP039143.1. Accessed 20 Jun 2021.
42. Ishizawa H, Kuroda M, Inoue D, Ike M. Complete Genome Sequences of Two Plant Growth-Inhibiting Bacteria, Acinetobacter ursingii M3 and Asticcacaulis excentricus M6, Isolated from Duckweed (Lemna minor). Microbiol Resour Announc. 2018;7(12).
43. Liu Z, Liu N, Feng Y, Wang A, Song D. Acinetobacter venetianus strain TUST-DM21 chromosome, complete genome. 2020. https://www.ncbi.nlm.nih.gov/nuccore/CP060161.1. Accessed 20 Jun 2021.
44. Hu Y, Feng Y, Qin J, Radolfova-Krizova L, Maixnerova M, Zhang X, et al. Acinetobacter wuhouensis sp. nov., isolated from hospital sewage. 2017. https://www.ncbi.nlm.nih.gov/nuccore/CP031716.1. Accessed 20 Jun 2021.
45. WTSI. Actinobacillus equuli strain NCTC9435 genome assembly, chromosome: 1. 2018. https://www.ncbi.nlm.nih.gov/nuccore/LR134486.1. Accessed 20 Jun 2021.
46. Che Y, Zhou L. Actinobacillus indolicus strain AIFJ1607 chromosome, complete genome. 2019. https://www.ncbi.nlm.nih.gov/nuccore/CP038145.1. Accessed 20 Jun 2021.
47. WTSI. Actinobacillus lignieresii strain NCTC10568 genome assembly, chromosome: 1. 2018. https://www.ncbi.nlm.nih.gov/nuccore/LR134169.1. Accessed 20 Jun 2021.
48. Park B-S, Lee N. Actinobacillus pleuropneumoniae Genome sequencing and assembly. 2017. https://www.ncbi.nlm.nih.gov/nuccore/CP022715.1. Accessed 20 Jun 2021.
49. Meng X-l. Actinomyces sp. nov. isolated from plateau pika in China. 2017. https://www.ncbi.nlm.nih.gov/nuccore/CP020468.1. Accessed 20 Jun 2021.
50. WTSI. Actinomyces israelii strain NCTC12972 genome assembly, chromosome: 1. 2018. https://www.ncbi.nlm.nih.gov/nuccore/LR134357.1. Accessed 20 Jun 2021.
51. Nambu T. Genome sequence of Actinomyces naeslundii strain ATCC 27039. 2016. https://www.ncbi.nlm.nih.gov/nuccore/AP017894.1. Accessed 20 Jun 2021.
52. Sproer C, Gronow S, Severitt S, Schroder I, Tallon L, Sadzewicz L, et al. FDA dAtabase for Regulatory Grade micrObial Sequences (FDA-ARGOS): Supporting development and validation of Infectious Disease Dx tests. 2020. https://www.ncbi.nlm.nih.gov/nuccore/CP065983.1. Accessed 20 Jun 2021.
53. URMITE. Actinomyces sp. Marseille-P2985 strain Marseille-P2985T genome assembly, chromosome: contig00001. 2016. https://www.ncbi.nlm.nih.gov/nuccore/LT635457.1. Accessed 20 Jun 2021.

54. Kanesaki Y, Murakami S, Sekigawa Y, Kobayashi T, Torii Y, Ishige T, et al. Emended Description of Actinomyces denticolens and Actinomyces suis that Indicates the Possibility of the Same Species as Actinomyces denticolens. 2016. https://www.ncbi.nlm.nih.gov/nuccore/AP017896.1. Accessed 20 Jun 2021.
55. Kristiansen R, Dueholm MS, Bank S, Nielsen PH, Karst SM, Cattoir V, et al. Complete Genome Sequence of Actinobaculum schaalii Strain CCUG 27420. 2014. https://www.ncbi.nlm.nih.gov/nuccore/CP008802.1. Accessed 20 Jun 2021.
56. Kim DU, Kim KW, Kang MS, Kim JY, Jang JH, Kim MK. Adhaeribacter swui sp. nov., isolated from wet mud. 2020. https://www.ncbi.nlm.nih.gov/nuccore/CP055156.1. Accessed 20 Jun 2021.
57. Wubbeler JH, Hiessl S, Schuldes J, Thurmer A, Daniel R, Steinbuchel A. Unravelling the complete genome sequence of Advenella mimigardefordensis strain DPN7T and novel insights in the catabolism of the xenobiotic polythioester precursor 3,3'-dithiodipropionate. 2012. https://www.ncbi.nlm.nih.gov/nuccore/CP003915.1. Accessed 20 Jun 2021.
58. Zhang G. New members of the family Aerococcaceae. 2020. https://www.ncbi.nlm.nih.gov/nuccore/CP065955.1. Accessed 20 Jun 2021.
59. Carkaci D, Dargis R, Nielsen XC, Skovgaard O, Fuursted K, Christensen JJ. Complete Genome Sequences of Aerococcus christensenii CCUG 28831T, Aerococcus sanguinicola CCUG 43001T, Aerococcus urinae CCUG 36881T, Aerococcus urinaeequi CCUG 28094T, Aerococcus urinaehominis CCUG 42038 BT, and Aerococcus viridans CCUG 4311T. 2016. https://www.ncbi.nlm.nih.gov/nuccore/CP014159.1. Accessed 20 Jun 2021.
60. Kerrigan L, Long C, Tallon L, Sadzewicz L, Vavikolanu K, Mehta A, et al. FDA dAtabase for Regulatory Grade micrObial Sequences (FDA-ARGOS): Supporting development and validation of Infectious Disease Dx tests. 2019. https://www.ncbi.nlm.nih.gov/nuccore/CP046334.1. Accessed 20 Jun 2021.
61. Varghese N, Submissions S. Aeromicrobium choanae strain 9H-4 genome assembly, chromosome: I. 2017. https://www.ncbi.nlm.nih.gov/nuccore/LT796768.1. Accessed 20 Jun 2021.
62. Zhou D. Complete sequence of chromosome of strain of 1607-10029. 2020. https://www.ncbi.nlm.nih.gov/nuccore/CP047981.1. Accessed 20 Jun 2021.
63. Cao G, Fu,J., Zhong C. Complete genome of Aeromonas media MC64. 2020. https://www.ncbi.nlm.nih.gov/nuccore/CP047963.1. Accessed 20 Jun 2021.
64. Zhou W, Chen Q. OXA-830, a novel chromosomally encoded expanded-spectrum class D beta-lactamase in Aeromonas simiae. 2019. https://www.ncbi.nlm.nih.gov/nuccore/CP040449.1. Accessed 20 Jun 2021.
65. Roh HJ, Kim DH. Whole genome analysis of multi-drug resistant Aeromonas veronii isolated from discus (Symphysodon discus). 2018. https://www.ncbi.nlm.nih.gov/nuccore/CP028133.1. Accessed 20 Jun 2021.
66. Daifuku T, Yoshida T, Kitamura T, Kawaichi S, Inoue T, Nomura K, et al. Variation of the virus-related elements within syntenic genomes of the hyperthermophilic Archaeon Aeropyrum. Appl Environ Microbiol. 2013;79(19):5891-8.

67. Kawarabayasi Y, Hino Y, Horikawa H, Yamazaki S, Haikawa Y, Jin-no K, et al. Complete genome sequence of an aerobic hyper-thermophilic crenarchaeon, Aeropyrum pernix K1. 1998. https://www.ncbi.nlm.nih.gov/nuccore/BA000002.3. Accessed 20 Jun 2021.
68. Watanabe M, Kojima H, Fukui M. Aerosticca soli gen. nov., sp. nov., an aerobic gammaproteobacterium isolated from crude oil-contaminated soil. 2018. https://www.ncbi.nlm.nih.gov/nuccore/AP018560.1. Accessed 20 Jun 2021.
69. Varghese N, Submissions S. Afipia sp. GAS231 genome assembly, chromosome: I. 2016. https://www.ncbi.nlm.nih.gov/nuccore/LT629703.1. Accessed 20 Jun 2021.
70. Ling S, Du Z. The whole genome sequence of Agarilytica rhodophyticola 017. 2017. https://www.ncbi.nlm.nih.gov/nuccore/CP020038.1. Accessed 20 Jun 2021.
71. Zhang P, Rui J, Mao X. The complete genome sequence of Agarivorans gilvus WH0801, a novel agarase-producing bacterium isolated from seaweed. 2015. https://www.ncbi.nlm.nih.gov/nuccore/CP013021.1. Accessed 20 Jun 2021.
72. Stacy A, Rumbaugh KP, Whiteley M. Tn-seq of a polymicrobial infection. 2015. https://www.ncbi.nlm.nih.gov/nuccore/CP012959.1. Accessed 20 Jun 2021.
73. WTSI. Aggregatibacter aphrophilus strain NCTC5906 genome assembly, chromosome: 1. 2018. https://www.ncbi.nlm.nih.gov/nuccore/LR134327.1. Accessed 20 Jun 2021.
74. Xiang T, Li Y, Tong X, Wu P, Lian C, Chen Z, et al. Complete genome sequence and characterization of cucumopine-type Agrobacterium rhizogenes strain K599 (NCPPB2659). 2018. https://www.ncbi.nlm.nih.gov/nuccore/CP019701.2. Accessed 20 Jun 2021.
75. Haryono M, Lin Y-C, Lai E-M, Kuo C-H. Complete genome sequence of Agrobacterium tumefaciens CFBP6625. 2019. https://www.ncbi.nlm.nih.gov/nuccore/CP039910.1. Accessed 20 Jun 2021.
76. Schuster SC. The complete genome of bacterial strain SGAirxxxx. 2018. https://www.ncbi.nlm.nih.gov/nuccore/CP027942.1. Accessed 20 Jun 2021.
77. Corretto E, Antonielli L, Sessitsch A, Compant S, Gorfer M, Kuffner M, et al. Agromyces aureus sp. nov., isolated from the rhizosphere of Salix caprea L. grown in a heavy-metal-contaminated soil. 2016. https://www.ncbi.nlm.nih.gov/nuccore/CP013979.1. Accessed 20 Jun 2021.
78. Koehorst J. Akkermansia glycaniphila isolate APytT genome assembly, chromosome: I. 2016. https://www.ncbi.nlm.nih.gov/nuccore/LT629973.1. Accessed 20 Jun 2021.
79. EMG. Akkermansia muciniphila isolate MGYG-HGUT-02454 genome assembly, chromosome: 1. 2019. https://www.ncbi.nlm.nih.gov/nuccore/LR699001.1. Accessed 20 Jun 2021.
80. Sun J-Q, Xu L. Verticiella alkalisoli sp. nov., isolated from alkaline-saline soil. 2019. https://www.ncbi.nlm.nih.gov/nuccore/CP041732.1. Accessed 20 Jun 2021.
81. Mahjoubi M, Aliyu H, Vikram S, Cappello S, Najjari A, Cowan DA, et al. Genome Sequence of the Petroleum-Degrading Alcaligenes aquatilis Strain BU33N isolated from petroleum contaminated sites in Tunisia. 2017. https://www.ncbi.nlm.nih.gov/nuccore/CP022390.1. Accessed 20 Jun 2021.

82. Lei L. Alcaligenes faecalis strain J481 chromosome, complete genome. 2018. https://www.ncbi.nlm.nih.gov/nuccore/CP032521.1. Accessed 20 Jun 2021.
83. Lai Q, Li W, Shao Z. Complete genome sequence of Alcanivorax dieselolei type strain B5. J Bacteriol. 2012;194(23):6674.
84. Lai Q, Shao Z. Genome sequence of an alkane-degrading bacterium, Alcanivorax pacificus type strain W11-5, isolated from deep sea sediment. J Bacteriol. 2012;194(24):6936.
85. Fu X, Lai Q, Dong C, Wang W, Shao Z. Complete genome sequence of Alcanivorax xenomutans P40, an alkane-#degrading bacterium isolated from deep seawater. 2015. https://www.ncbi.nlm.nih.gov/nuccore/CP012331.1. Accessed 20 Jun 2021.
86. Sun C, Fu G, Zhang C, Wu M. Algibacter alginicilyticus strain HZ22, complete genome. 2015. https://www.ncbi.nlm.nih.gov/nuccore/CP012898.1. Accessed 20 Jun 2021.
87. Chen X. Complete genome sequence of Alicycliphilus denitrificans DP3. 2020. https://www.ncbi.nlm.nih.gov/nuccore/CP051298.1. Accessed 20 Jun 2021.
88. Wiegand S, Jogler M, Boedeker C, Pinto D, Vollmers J, Rivas-Marin E, et al. Deep-cultivation of Planctomycetes and their phenomic and genomic characterization uncovers novel biology. 2019. https://www.ncbi.nlm.nih.gov/nuccore/CP036265.1. Accessed 20 Jun 2021.
89. Miller WG, Yee E. Complete genome sequencing of Campylobacter and Arcobacter type strains. 2020. https://www.ncbi.nlm.nih.gov/nuccore/CP053837.1. Accessed 20 Jun 2021.
90. Hjerde E. Aliivibrio wodanis genome assembly AWOD1, chromosome : 2. 2014. https://www.ncbi.nlm.nih.gov/nuccore/LN554847.1. Accessed 20 Jun 2021.
91. Sakamoto M, Ikeyama N, Ogata Y, Suda W, Iino T, Hattori M, et al. Alistipes communis sp. nov., Alistipes dispar sp. nov. and Alistipes onderdonkii subsp. vulgaris subsp. nov., isolated from human faeces, and creation of Alistipes onderdonkii subsp. onderdonkii subsp. nov. 2019. https://www.ncbi.nlm.nih.gov/nuccore/AP019734.1. Accessed 20 Jun 2021.
92. Weissgerber T, Zigann R, Bruce D, Chang YJ, Detter JC, Han C, et al. Complete genome sequence of Allochromatium vinosum DSM 180(T). 2010. https://www.ncbi.nlm.nih.gov/nuccore/CP001897.1. Accessed 20 Jun 2021.
93. Varghese N, Submissions S. Allokutzneria albata strain DSM 44149 genome assembly, chromosome: I. 2016. https://www.ncbi.nlm.nih.gov/nuccore/LT629701.1. Accessed 20 Jun 2021.
