# Supplementary material for: CrisprVi: a software for visualizing and analyzing CRISPR sequences of prokaryotes
Source: BMC Bioinformatics. 2022 May 11;23(Suppl 3):172. doi: 10.1186/s12859-022-04716-9 (PMC9128103; doi:10.1186/s12859-022-04716-9)
Supplement: Supplementary file 3 — Additional file 3. Fig. S1: Snapshot of visualizing CRISPRs of 80 strains on CrisprVi. [file 12859_2022_4716_MOESM3_ESM.pdf]

X	enome1_AP019695.	0’.	0.	0’.	1.	0’.	2.	0’.	3.	0’.	4.	0’.	5.	0’.	6.	0’.	7.	0’.	8.
X	enome10_CP039845	1’.	18.	2’.	19.	2’.	20.	3’.	21.	4’.	22.	5’.	23.	6’.	24.	7’.	8’.	25.	8’.
X	nome100_LT629701	10’.	50.	11’.	51.	12’.	52.	11’.	53.	11’.	54.	11’.	55.	11’.	56.	11’.	57.	11’.	58.
X	enome11_AP011122	37’.	93.	37’.	94.	37’.	95.	37’.	96.	37’.	97.	37’.	98.	37’.	99.	37’.	100.	37’.	101.
X	enome12_CP023657	39’.	116.	40’.	117.	40’.	118.	40’.	119.	40’.	120.	40’.	121.	40’.	122.	41’.	123.	40’.	124.
X	enome13_CP030871	42’.	146.	42’.	147.	42’.	148.	42’.	149.	42’.	150.	42’.	151.	43’.	152.	42’.	153.	42’.	154.
X	enome15_F0681347	45’.	159.	45’.	160.	45’.	161.	45’.	162.	45’.	163.	45’.	164.	45’.	165.	45’.	166.	45’.	167.
X	enome17_CP026124	48’.	195.	49’.	196.	49’.	197.	50’.	198.	50’.	199.	50’.	200.	50’.	201.	50’.	202.	50’.	203.
X	enome18_CP001859	56’.	282.	56’.	283.	56’.	284.	56’.	285.	56’.	286.	56’.	287.	56’.	288.	56’.	289.	56’.	290.
X	enome19_CP045482	60’.	343.	60’.	344.	60’.	345.	60’.	346.	60’.	347.	60’.	348.	60’.	349.	60’.	350.	60’.	351.
X	enome20_CP000690	66’.	451.	66’.	452.	66’.	453.	66’.	454.	66’.	455.	66’.	456.	66’.	457.	66’.	458.	66’.	459.
X	enome21_CP025198	71’.	490.	72’.	491.	73’.	492.	74’.	493.	75’.	494.	76’.	495.	77’.	496.	77’.	497.	77’.	498.
X	enome22_CP005986	91’.	693.	92’.	694.	92’.	695.	93’.	94’.	696.	95’.	697.	94’.	698.	94’.	699.	96’.	700.	97’.
X	enome24_CP028292	103’.	712.	104’.	713.	105’.	714.	106’.	715.	107’.	716.	107’.	717.	107’.	718.	107’.	719.	107’.	720.
X	enome25_CP028290	108’.	752.	108’.	753.	108’.	754.	108’.	755.	108’.	756.	108’.	757.	108’.	758.	108’.	759.	108’.	760.
X	enome26_CP000539	115’.	805.	115’.	806.	115’.	807.	115’.	808.	115’.	809.	115’.	810.	115’.	115’.	811.	115’.	812.	115’.
X	enome27_CP001941	116’.	876.	117’.	877.	117’.	878.	117’.	879.	117’.	877.	117’.	880.	117’.	881.	117’.	882.	117’.	118’.
X	enome28_LN997846	119’.	897.	120’.	898.	121’.	899.	122’.	900.	123’.	901.	123’.	902.	123’.	903.	123’.	904.	123’.	905.
X	enome29_CP041035	124’.	956.	124’.	957.	124’.	958.	124’.	959.	124’.	960.	124’.	961.	124’.	962.	124’.	963.	124’.	964.
X	enome3_AP023326.	130’.	1007.	130’.	1008.	130’.	1009.	130’.	1010.	130’.	1011.	130’.	1007.	130’.	1012.	130’.	1013.	130’.	1014.
X	enome30_CR543861	136’.	1052.	137’.	1053.	137’.	1054.	137’.	1055.	138’.	1056.	137’.	1057.	137’.	139’.	1058.	139’.	1059.	139’.
X	enome31_CP066121	144’.	1162.	144’.	1162.	144’.	1163.	144’.	1164.	144’.	1165.	144’.	1166.	145’.					
X	enome32_CP032134	146’.	1167.	147’.	1168.	147’.	1169.	148’.	1170.	149’.	1171.	149’.	1172.	149’.	1173.	147’.	1174.	147’.	1175.
X	enome33_AP014630	150’.	1180.	150’.	1181.	150’.	1182.	150’.	1183.	150’.	1184.	150’.	1185.	150’.	1186.	150’.	1187.	150’.	1188.
