# Supplementary figures and images for: CrisprVi: a software for visualizing and analyzing CRISPR sequences of prokaryotes
Source: BMC Bioinformatics. 2022 May 11;23(Suppl 3):172. doi: 10.1186/s12859-022-04716-9 (PMC9128103; doi:10.1186/s12859-022-04716-9)

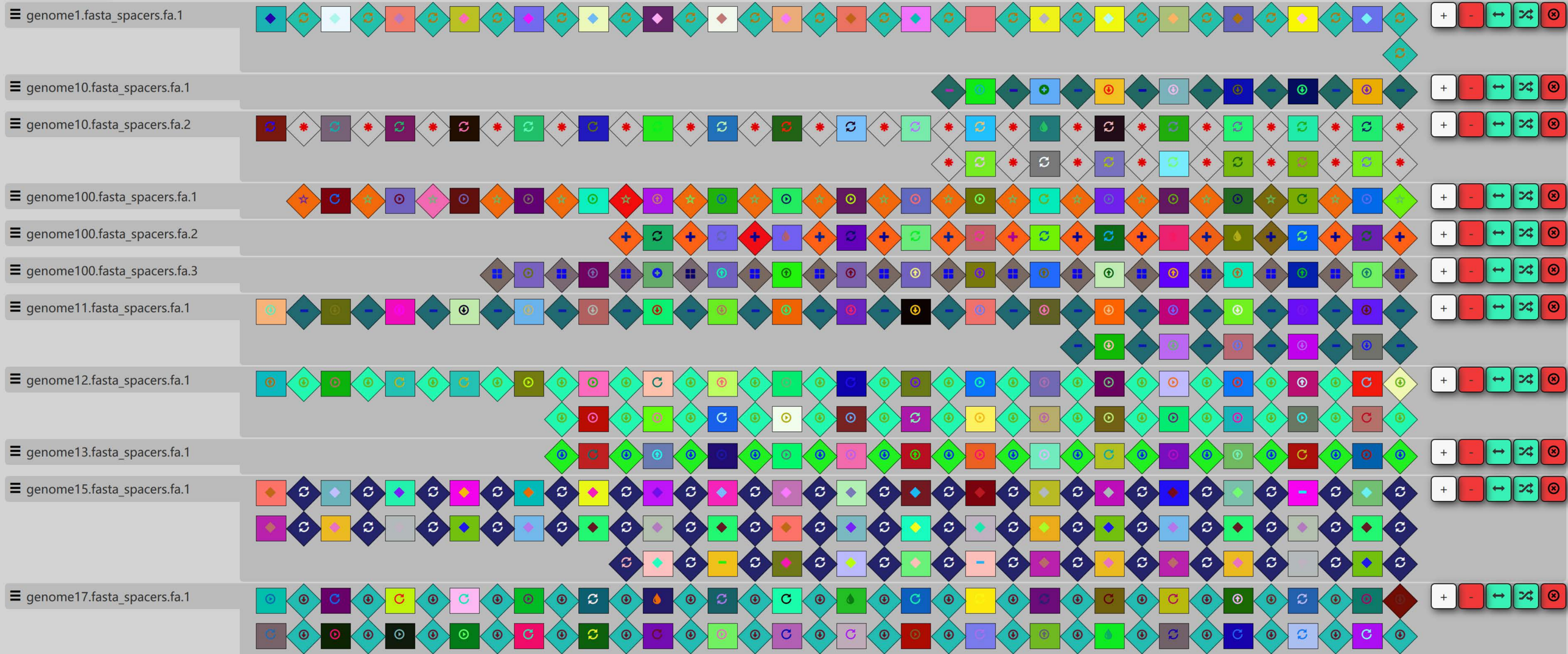

Supplement: Supplementary file 4 — Additional file 4. Fig. S2: Snapshot of visualizing CRISPRs of 80 strains on CRISPRviz. [file 12859_2022_4716_MOESM4_ESM.pdf]
